# Potassium suppresses allosteric activation of ZAP-70-dependent T cell receptor signaling

**DOI:** 10.1016/j.jbc.2026.113083

**Published:** 2026-04-28

**Authors:** Swarnendu Roy, Soumee SenGupta, Kaustav Gangopadhyay, Sudipta Majumder, Jibitesh Das, Anushka Sinha, Prosad Kumar Das, Bidisha Sinha, Rahul Das

**Affiliations:** 1 Department of Biological Sciences, Indian Institute of Science Education and Research Kolkata, Mohanpur, India; 2 Centre for Advanced Functional Materials, Indian Institute of Science Education and Research Kolkata, Mohanpur, India

**Keywords:** T cell receptor, tyrosine kinase, cell signaling, potassium, ZAP-70

## Abstract

Ionic imbalance in the tumor microenvironment alters the function of tumor-infiltrating T lymphocytes. High extracellular K^+^ suppresses T cell function by negatively regulating T cell receptor (TCR) signaling. The mechanism of how monovalent cations regulate T lymphocyte function is unknown. Here, we present a mechanism that explains how cellular potassium dynamics regulate TCR function. At rest, high intracellular K^+^ uncouples allosteric recruitment of ZAP-70, a key signaling module, to the TCR complex. Elevated K^+^ concentration imparts a higher thermodynamic penalty on the binding of the ZAP-70 regulatory module to the phosphotyrosine residues in the ITAM motifs of the CD3 chain. Our data suggest that K^+^ functions as a key allosteric modulator, stabilizing the autoinhibited conformation of ZAP-70. Thus, it prevents spontaneous TCR activation in the resting state. Formation of the antigen–TCR complex induces K^+^ efflux, leading to spontaneous recruitment of ZAP-70 to the TCR. Increasing extracellular K^+^ concentration perturbs K^+^ efflux and slows ZAP-70 recruitment to the TCR complex, even upon antigen binding. Impaired ZAP-70 activation partially dampens TCR signaling, thereby altering downstream signaling. In contrast, the regulatory module in the paralogous kinase Syk, which is expressed in B cells, is insensitive to potassium concentration. At elevated K^+^ concentration, the interaction between the Syk regulatory module and phosphorylated ITAM motifs remains unaltered. We conclude that K^+^ dynamics are integral to T cell ligand discrimination and fundamental to turning off the signaling during T cell quiescence.

Monovalent salts of sodium (Na^+^) and potassium (K^+^) ions are the key regulators of T cell effector function in the tumor microenvironment (TME) (1, 2, 3, 4, 5). Counter-intuitively, K^+^ regulates T cell effector function in the TME differently than Na^+^. The elevated Na^+^ enhances the effector function of tumor-infiltrating T lymphocytes (TIL), whereas high K^+^ in the TME suppresses the TIL function. Both monovalent cations influence TIL function by altering cell metabolism and T-cell receptor (TCR) signaling. For example, elevated extracellular K^+^ induces functional caloric restriction of TIL by limiting glucose uptake while promoting T cell stemness (1). Additionally, high extracellular [K^+^]_e_ in TME suppresses TCR signaling by dephosphorylating downstream signaling modules (1, 2, 6). In contrast, elevated Na^+^ aids phosphorylation of TCR signaling modules and glutamine consumption, boosting the effector function of CD8^+^ T cells (3, 4). Moreover, a high-salt diet (containing NaCl) promotes CD4^+^ T cell differentiation to T helper 17 (Th17). It impairs T regulatory (T_reg_) cells’ suppressive function, linking high dietary sodium to various autoimmune disorders (7, 8). The molecular basis of why Na^+^ and K^+^ antithetically regulate TCR signaling is an open question.

Engagement of TCR with the antigen presented through the major histocompatibility complex (MHC) of the antigen-presenting cells initiates T cell signaling (Fig. 1*A*) (9, 10). Antigen binding induces restructuring of the TCR microcluster, forming an immune synapse (IS) (11, 12). Initially, two tyrosine kinases, a Src family kinase, Lck, and a Syk family kinase, ZAP-70, are recruited to the TCR (13). Lck phosphorylates multiple tyrosine residues in the immunoreceptor tyrosine-based activation motifs (ITAM) in the CD3 chains (13, 14). The ZAP-70 is then spontaneously recruited to the TCR microcluster by binding to the doubly phosphorylated tyrosine residues in the ITAM (ITAM-Y_2_P) motifs (15, 16, 17). Subsequent autophosphorylation of a tyrosine residue in the activation loop of the kinase domain activates the ZAP-70 (18, 19). The activated ZAP-70 then phosphorylates the scaffold protein LAT, which, in turn, recruits phospholipase C (PLC), eventually triggering the downstream Ca^2+^ influx, Akt-mTOR, and Erk signaling pathways (Fig. 1*A*) (9, 13). Initially, the Ca^2+^ is released from the intracellular stores (ER) triggered by inositol (1, 4, 5)-trisphosphate [Ins (1, 4, 5)P3]. Depletion of the internal Ca^2+^ source activates voltage-independent Ca^2+^ release-activated Ca^2+^ (CRAC) channels, resulting in sustained Ca^2+^ influx (20). Two potassium channels, shaker-related voltage-gated potassium channels (Kv1.3) and Ca^2+^ activated potassium channel (K_Ca_3.1), are the primary regulators of membrane potential and calcium flux in T lymphocytes (21, 22, 23). The Kv1.3 colocalized to the TCR microcluster within minutes of antigen binding (21, 24, 25, 26). The K^+^ efflux thus helps sustain Ca^2+^ influx and propagation of TCR signaling downstream (25, 27, 28, 29). Selective blocking of Kv1.3 channels suppresses the immune response and inhibits anti-CD3-dependent T cell proliferation, supporting a fundamental role of intracellular K^+^ in T cell physiology (22, 30, 31). The potassium channel blockers are thus effective therapeutic molecules for treating diverse autoimmune diseases, such as rheumatoid arthritis (RA), multiple sclerosis, and type 1 diabetes (32, 33, 34, 35). However, the molecular mechanism of how intracellular K^+^ regulates TCR activation and ligand discrimination remains elusive.

**Figure 1:**
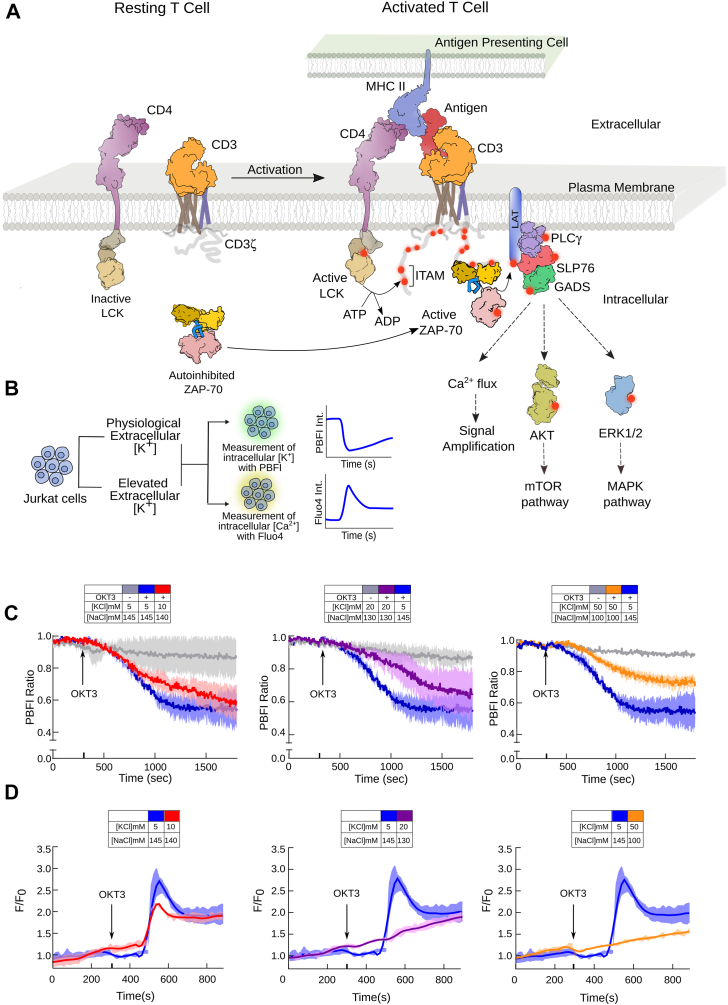
**Measurement of TCR-induced potassium efflux and calcium influx in activated Jurkat E6.1 T cells.***A*, a schematic representation of the TCR signaling pathway. The signaling modules in the TCR complex are labeled. *B*, the experimental outline for estimating intracellular potassium and calcium levels in Jurkat E6.1 T cells. The T cells are activated with an anti-CD3 antibody (OKT3) in different extracellular KCl concentrations. The intracellular potassium level is measured from the ratio of PBFI fluorescence intensity measured at λ_ex_ of 340 nm and 380 nm, respectively, and λ_em_ of 505 nm. The intracellular calcium level is determined from the change in fluorescence intensity of Fluo4 dyes when measured at λ_ex_ of 495 nm and λ_em_ of 516 nm. *C*, potassium efflux is measured in Jurkat E6.1 T cells at the indicated extracellular KCl concentration. The T cells were activated with an anti-CD3 antibody (OKT3) at the indicated time. The change in PBFI fluorescence intensity is plotted over time. *D*, the cytosolic calcium level is determined from the plot of normalized Fluo4 intensity as a function of time. At the indicated time, the T cells were activated in the presence of indicated KCl concentration with OKT3. The Fluo4 intensity for the activated (F) T cells is normalized against the Fluo4 intensity measured for the inactive (F_o_) cells. In *panel C* and *D*, the solid lines represent the mean from three independent experiments, and the area fill denotes the SD. In each experimental series, Jurkat E6.1 T cells at the same passage were activated (on the same day) under the indicated salt conditions. All data were plotted using GraphPad PrismVer9.5.1. The schematics and icons were made using Inkscape Ver 1.4. See Fig. S1.

Kinetic proofreading enables T cells to discriminate between self and non-self-antigens by calibrating the TCR response to antigen binding (36). The half-life of the antigen-receptor complex, the subsequent delay in LAT phosphorylation by ZAP-70, and the time taken for switching Ca^2+^ influx are crucial for TCR selectivity and sensitivity (37, 38, 39, 40). The short-lived self-antigen–TCR complex will be disassembled before the downstream enzymes are recruited to the IS. At the same time, only the long-lived antigen-TCR complex will initiate a downstream TCR response. The mechanism by which ionic imbalance interferes with the kinetic proofreading of TCR is unknown.

This paper studied the TCR response to antigen binding across varying salt concentrations. We presented a molecular mechanism explaining how ionic imbalance uncouples antigen binding from downstream signaling. We observed that the K^+^ efflux in response to TCR stimulation is sensitive to extracellular salt composition. High extracellular [K^+^]_e_ alters K^+^ efflux in Jurkat cells upon TCR stimulation. An elevated intracellular [K^+^]_i_ slows ZAP-70 recruitment to the IS, switching off the Ca^2+^ influx and downstream signaling. Our data suggest that K^+^ is a key allosteric regulator that, at higher intracellular concentrations, uncouples ZAP-70 ligand binding at the TCR complex. We proposed that intracellular K^+^ dynamics are integral to TCR ligand discrimination and to maintaining basal signaling off.

## Results and discussion

### High extracellular potassium prevents K^+^ efflux and Ca^2+^ influx upon TCR stimulation

At rest, the negative membrane potential of the T cell is maintained by sustaining a high intracellular [K^+^]_i_ concentration (∼130 mM) (1, 41). The engagement of the TCR and antigen induces Ca^2+^ influx, leading to depolarization of the cell membrane. The negative membrane potential is re-established by effluxing K^+^ out of the cell, mainly by Kv1.3 channels (21, 24). An increase of extracellular [K^+^]_e_ to 20 to 40 mM in the TME (from ∼ 5 mM [K^+^]_e_ in serum) marginally increases intracellular [K^+^]_i_ concentration (140–150 mM) in TIL (1). It is unclear why a subtle increase in intracellular [K^+^]_i_ levels affects T cell function. The mechanism by which ionic imbalance in the TME modulates K^+^ efflux and downstream Ca^2+^ influx upon T cell stimulation is unclear, primarily due to the lack of direct measurement of K^+^ efflux.

Here, we measure K^+^ efflux and Ca^2+^ influx in activated Jurkat E6.1 T cells in media containing various ionic compositions (Fig. 1, *B*–*D*). The change in intracellular [K^+^]_i_ or [Ca^2+^]_i_ concentrations was determined from the change in PBFI-AM (42, 43) or Fluo4 (44) fluorescence intensity, respectively, after activating the T cells with an anti-CD3 monoclonal antibody (OKT3). A decrease in PBFI-AM fluorescence intensity indicates K^+^ efflux, whereas an increase in Fluo4 fluorescence intensity indicates Ca^2+^ influx. We observed that stimulating Jurkat E6.1 cells decreased intracellular [K^+^]_i_ and induced Ca^2+^ flux (Fig. 1, *C* and *D*). At a physiological [K^+^]_e_ concentration (5 mM), K^+^ efflux begins shortly after TCR activation (Fig. 1*C*), and Ca^2+^ influx was observed (Fig. 1*D*). However, higher extracellular [K^+^]_e_ concentration perturbs the TCR-dependent K^+^ efflux and dampens subsequent Ca^2+^ signaling. At a KCl concentration of 20 mM or higher, the Ca^2+^ flux is completely attenuated (Figs. 1*D*, S1*C*, Tables S2, and S3). Higher extracellular [K^+^]_e_ reduces the rate of K^+^ efflux and maintains a relatively higher intracellular K^+^ level compared to the T cells activated under physiological [K^+^]_e_ levels (Figs. 1*C* and S1, *D* and *E*). We ask if maintaining a higher intracellular potassium concentration can decouple the antigen binding from downstream Ca^2+^ signaling.

Kv1.3 potassium channel is essential for antigen-dependent T lymphocyte activation and proliferation (21, 24, 31). We observe that blocking the Kv1.3 channels with clofazimine (45) prevents K^+^ efflux, thereby retaining high intracellular potassium levels even after TCR activation (Fig. S1, *A*–*E*, Tables S2, and S3). Inhibiting the Kv1.3 channel prevents Ca^2+^ influx upon antigen binding (Fig. S1*A*) (23, 27), indicating that high intracellular [K^+^]_i_ may suppress T lymphocyte function by decoupling the antigen binding and Ca^2+^ flux. It is no wonder that several Kv1.3 inhibitors, which also suppress T cell function, have the potential to treat multiple autoimmune disorders (32, 33, 34, 35, 46, 47). However, prolonged treatment with Kv1.3 blockers has previously been shown to inhibit the thymic development of T lymphocytes (30). We speculate that intracellular K^+^ dynamics may play a fundamental role in regulating T cell function (Fig. S1*F*). High intracellular [K^+^]_i_ attenuates TCR downstream signaling upon antigen binding. Therefore, we next investigated the effect of high [K^+^]_i_ levels on early TCR signaling, which couples Ca^2+^ influx to antigen binding (Fig. 1*A*).

### High extracellular potassium interferes with the ZAP-70-dependent TCR signaling

**Figure 2:**
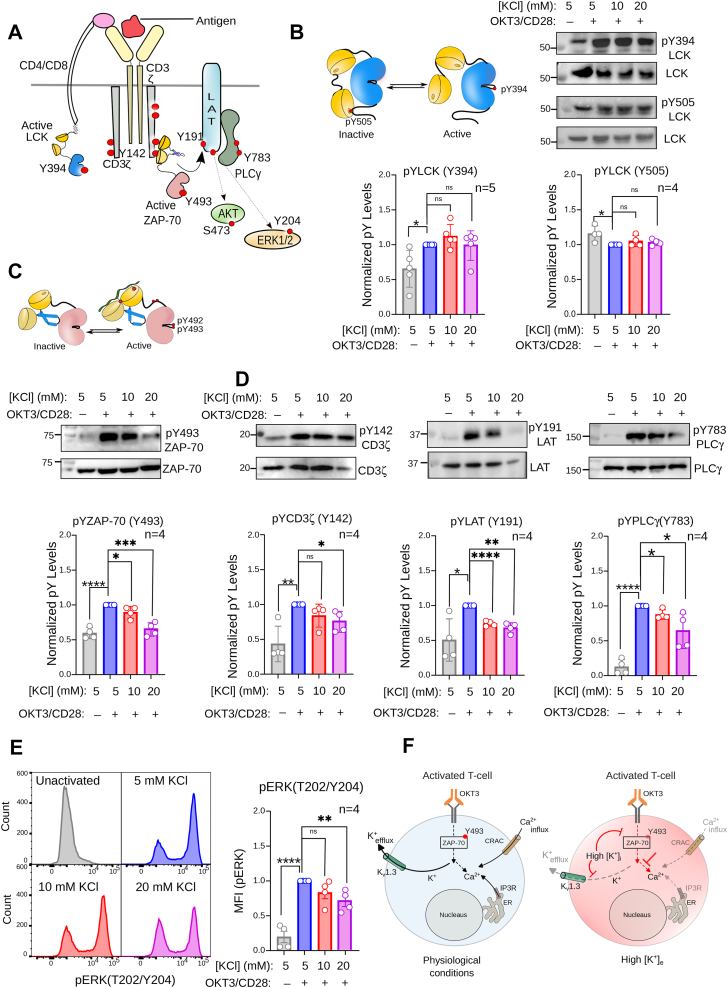
**Intracellular potassium levels regulate ZAP-70-dependent TCR signaling.***A*, schematic representation of the activated TCR signaling pathway. The signaling modules evaluated in this study are labeled. *B*, representative immunoblot showing the phosphorylation of Lck Y505 or Y394, corresponding to inactive or active states, respectively. The Jurkat E6.1 T cells were stimulated with OKT3/anti-CD28 antibodies at the indicated concentrations of extracellular KCl and then immunoblotted with the indicated anti-pY and anti-protein antibodies. The *bottom panel* shows the densitometric analysis of the immunoblots. Bar graphs represent the fold changes in phospho-tyrosine levels of specific proteins in the TCR signaling modules at the indicated potassium concentration. For pLCK (Y394) from *left to right*: *p* = 0.0179; *p* = 0.1762; *p* = 0.8947, for pLCK (Y505) from *left to right*: *p* = 0.0306; *p* = 0.2348; *p* = 0.1841. *C*, representative immunoblot showing the phosphorylation of ZAP-70 Y493, corresponding to the active state. The Jurkat E6.1 T cells were stimulated as explained in *Panel B*. The *bottom panel* is the densitometric analysis of the immunoblots. For pZAP-70 (Y493) from *left to right*: *p* < 0.0001; *p* = 0.0427; *p* = 0.0003. *D*, representative immunoblots showing the phosphorylation of various TCR signaling modules. The *bottom panel* densitometric analysis of the immunoblots. For pCDζ from *left to right*: *p* = 0.0044; *p* = 0.1016; *p* = 0.0175, for pLAT (Y191) from left to right: *p* = 0.0172; *p* < 0.0001; *p* = 0.0012, for pPLCγ (Y783) from *left to right*: *p* < 0.0001; *p = 0.0104*; *p* = 0.0029. *E*, representative flow cytometry histograms of phospho-ERK1/2 (T202/Y204) in the unactivated and activated Jurkat E6.1 T cells at the indicated extracellular potassium concentration. Bar graphs on the right show the fold changes of ERK half phosphorylation in stimulated Jurkat cells at the indicated potassium concentration in the extracellular media. For pERK (T202/Y204) from *left to right*: *p* < 0.0001; *p* = 0.1102; *p* = 0.0082. *F*, schematic representation summarizing the effect of elevated [K^+^]_i_ on the TCR signaling. *Black solid* or *dotted arrows* indicate the pathways connecting TCR stimulation to K^+^ efflux and Ca^2+^ influx, respectively. *Red dotted or solid blunt arrows* indicate the pathway inhibited in the presence of elevated [K^+^]_e_ or clofazimine treatment. *C* and *E*, a statistical analysis of two-tailed Student’s t-tests was performed. Each data point represents the mean ± SD from three independent experiments. (ns = not significant; ∗*p* < 0.05; ∗∗*p* < 0.01; ∗∗∗*p* < 0.001; ∗∗∗∗*p* < 0.0001). All data were plotted using GraphPad PrismVer9.5.1. The flow cytometry data were analyzed using FlowJo Ver8. The schematics and icons were made using Inkscape Ver 1.4. See Fig. S2.

TCR lacks intrinsic catalytic activity. The signaling starts with the sequential recruitment of two tyrosine kinases, Lck and ZAP-70 (Fig. 2*A*) (13). Thus, we begin by probing the effect of excess extracellular [K^+^]_e_ on overall phosphorylation status in activated Jurkat E6.1 T cells. We observed that higher extracellular [K^+^]_e_ levels partially impaired total tyrosine phosphorylation in Jurkat E6.1 T cells compared with those activated under physiological [K^+^]_e_ concentrations (Fig. S2*A*). Our data indicate that potassium negatively regulates tyrosine kinase activation in a concentration-dependent manner. Therefore, we next focus on the effect of elevated [K^+^]_i_ on the activation of Lck and ZAP-70. The active and inactive conformations of Lck were probed by measuring the phosphorylation of two mutually exclusive tyrosine residues, Y394 and Y505 (Fig. 2*B*) (48). The phosphorylation of key tyrosine residues in the activation loop of Lck (Y394) and ZAP-70 (Y493) is a hallmark of the activated kinase (Fig. 2, *B* and *C*) (9). The phosphotyrosine stabilizes the active conformation of the enzyme by preventing the activation loop from folding back into the catalytic site (49). Unexpectedly, elevated extracellular [K^+^]_e_ did not inhibit Lck Y394 phosphorylation (Fig. 2*B*) (6). The phosphorylation levels of Y505, which stabilizes an inactive conformation of Lck, were lower in the stimulated T cells, even in the presence of elevated extracellular [K^+^]_e_. Thus, our data suggest that elevated [K^+^]_e_ does not perturb the balance between the inactive or active state of Lck in T cells. Instead, we observed that elevated extracellular [K^+^]_e_ inhibits ZAP-70 activation in a concentration-dependent manner (Fig. 2*C*). The phosphorylation of Y493 was partially, but significantly, reduced at 20 mM KCl compared to the physiological concentration of 5 mM. As anticipated, inhibiting ZAP-70 at higher [K^+^] partially reduces the phosphorylation of multiple downstream signaling modules, like LAT, PLCγ, Erk, and Akt (Figs. 2, *D* and *E* and S2*B*). To independently verify if higher intracellular [K^+^]_i_ inhibits ZAP-70 activation, we determined the Y493 phosphorylation in activated Jurkat E6.1 T cells treated with clofazimine (Fig. S2*C*). Clofazimine maintains higher intracellular [K^+^]_i_ (∼120–150 mM) (1) by blocking K^+^ efflux through Kv1.3 channels during TCR activation (Fig. S1, *B*, *D*, and *E*). We observed that clofazimine treatment completely inhibits ZAP-70 autophosphorylation, even after TCR stimulation, suggesting that high intracellular [K^+^]_i_ inhibits ZAP-70 activation, leading to inhibition of Ca^2+^ flux (Figs. 1*A* and S1*A*) and Erk phosphorylation (Fig. S2*D*). Together, our data suggest that high intracellular [K^+^]_i_ inhibits ZAP-70 activation, which in turn attenuates downstream signaling modules (Figs. 2*F* and S1*F*).

Since higher intracellular potassium does not perturb Lck activation, it is surprising that elevated extracellular [K^+^]_e_ marginally reduces the phosphorylation of tyrosine residues in the ITAM motifs of the CD3-ζ chain (Fig. 2*D*). Previous independent studies showed reduced CD3-ζ phosphorylation in the SKG mice bearing ZAP-70 ^W165C^ mutant, which impairs interaction between ZAP-70^W165C^ and CD3-ζ chain (50, 51). We speculate that the decreased tyrosine phosphorylation of CD3-ζ may be due to the dephosphorylation of ITAM motifs by phosphatases present in the IS, which is otherwise preserved by the ZAP-70 and ITAM-Y_2_P complex (52). Therefore, we next investigate how a higher intracellular [K^+^]_i_ concentration prevents interaction between ZAP-70 and CD3-ζ.

### Potassium allosterically regulates the interaction between ITAM-Y_2_P in the CD3 chain and the regulatory module of ZAP-70

**Figure 3:**
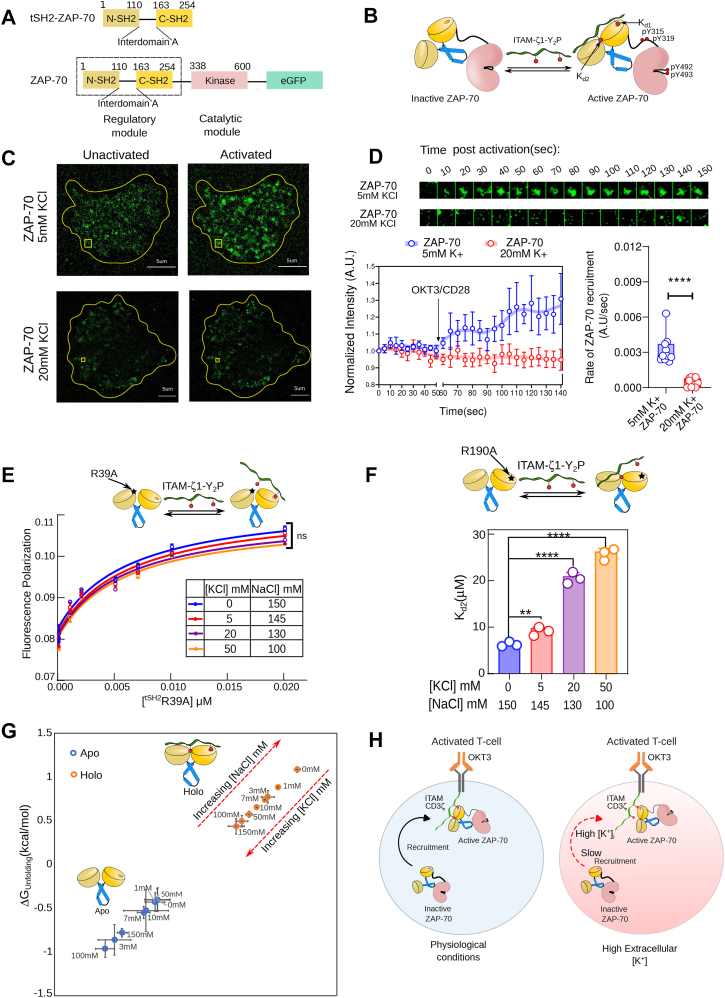
**Higher potassium concentration prevents ZAP-70 and CD3-ζ interaction in the TCR complex.***A*, a schematic representation of the domain architecture of ZAP-70 constructs used in this study. *B*, cartoon representation of the ZAP-70 activation model. *C*, representative TIRF microscopic image of unactivated (on the *left*) and activated (on the *right*) live Jurkat P116 T cell stably expressing the indicated ZAP-70-EGFP construct. The cells are activated with anti-CD3/anti-CD28 mAb at the indicated KCl concentration. The cell boundary is marked with a *yellow line*, and a *yellow box* indicates a region of the ZAP-70-EGFP cluster. *D*, the *top panel* represents a time-lapse montage of a representative ZAP-70-EGFP cluster after activation with OKT3/anti-CD28 mAb at the indicated extracellular KCl concentration. The *bottom panel* at the *left* is the plot of EGFP intensity as a function of time, measured from five ZAP-70 clusters (size: 10 pixels, 1pixel = 0.65 μm) in one live Jurkat P116 T cell. The *arrow* indicates the time point when OKT3/anti-CD28 mAb is added. Each point represents the average intensity of five ZAP-70 clusters, and the error bar denotes the SEM. The *red* and *blue solid lines* are guiding lines. The *bottom panel* on the right is the plot of the rate of change in EGFP intensity from the individual cells at the indicated KCl concentration. The error represents SD from ten cells (n = 10), *p* < 0.0001. *E*, the plot of fluorescence polarization as a function of tSH2 domain concentration, determined from the titration of the ^R39A^tSH2 mutant and ITAM-ζ1-Y_2_P tagged to Alexa Fluor488. Each data point is the mean ± SD from three independent experiments. The c*olored solid lines* represent the fitting to the first-order binding. *F*, the bar graph shows the *K*_*d*_ values for the N-SH2 phosphate-binding pocket and the ITAM-ζ1-Y_2_P interaction. The *K*_*d*_ represents the mean value from three independent ITC titrations of the ^R190A^tSH2 mutant and ITAM-ζ1-Y_2_P. *p* value: 5 mM KCl: = 0.0023, 20 mM KCl:<0.0001, 50 mM KCl:<0.0001 G) ΔG_unfolding_ for the *apo* and *holo* tSH2 domain of ZAP-70 is plotted at the indicated KCl concentration. The ΔG_unfolding_ is derived from the thermal denaturation profile measured using Circular Dichroism (CD) spectroscopy (Fig. S4*C*). The data points were rearranged diagonally by plotting ΔG_unfolding_ on both axes. Each data point represents the mean ± SD from three independent experiments. *H*, schematic representation summarizing the ZAP-70 recruitment to the plasma membrane in the presence of high [K^+^]_i_. *Black solid* or *dotted arrows* indicate the pathways connecting TCR stimulation to K^+^ efflux and Ca^2+^ influx, respectively. *Red dotted arrows* indicate the pathway inhibited in the presence of elevated [K^+^]_e_. *D* and *F*, a statistical analysis of two-tailed Student’s t-tests was performed. Central values and error bars represent mean ± SD (ns = not significant; ∗*p* < 0.05; ∗∗*p* < 0.01; ∗∗∗*p* < 0.001; ∗∗∗∗*p* < 0.0001). All data were plotted using GraphPad PrismVer9.5.1. The image analysis was done using Fiji Ver 1.54 m. The schematics and icons were made using Inkscape Ver 1.4. See Figs. S3 and S4.

ZAP-70 bridges a key kinetic bottleneck between the antigen-TCR complex and the LAT-based signalosome, which is essential for ligand discrimination (38). ZAP-70 is activated in two steps: first, the enzyme is recruited to the TCR complex, and then it is activated by phosphorylating multiple tyrosine residues by Lck or through autophosphorylation (53). Since higher intracellular [K^+^]_i_ does not perturb Lck activity (Fig. 2*B*), we focus on the recruitment of ZAP-70 to the TCR complex. The recruitment of ZAP-70 to the plasma membrane is mediated by the interaction between ITAM-Y_2_P in the CD3-ζ and the regulatory module of ZAP-70 (Fig. 3*A*) (17, 52). The ZAP-70 regulatory module consists of a tandem Src homology domain (tSH2) linked to the C-terminal kinase domain (Fig. 3, *A* and *B*). In the autoinhibited state, the tSH2 domain adopts an open conformation (Fig. 3*B*) (54). The binding of ITAM-Y_2_P remodels the tSH2 domain structure to a closed conformation, subsequently releasing the autoinhibition of the kinase domain (52, 55). We first investigate if increased intracellular [K^+^]_i_ levels modulated the interaction between ZAP-70 and CD3 at the plasma membrane. We measured the localization of the ZAP-70 tagged to EGFP to the plasma membrane using total internal reflection fluorescence (TIRF) microscopy following T cell stimulation (Figs. 3, *C* and *D* and S3). The TIRF image shows the formation of distinct EGFP clusters at the plasma membrane of the live Jurkat P116 cells activated in 5 mM or 20 mM KCl (Figs. 3*C* and S3, *A*, *B*, and *E*). Our image analysis revealed that the ZAP-70 clustering is significantly slower when T cells are activated in media containing 20 mM KCl compared to 5 mM KCl (Figs. 3*D* and S3*D*). We next investigate how higher intracellular potassium levels perturb ZAP-70 recruitment to the TCR.

The tSH2 domain of ZAP-70 consists of two SH2 domains, N-SH2 and C-SH2, connected by a helical linker (Fig. 3*A*). We have previously shown that ITAM-Y_2_P binds first to the C-SH2 phosphate-binding pocket (PBP) with a strong (nM) affinity (*K*_*d1*_), forming an encounter complex (40, 50). That, in turn, induces the tSH2 domain to adopt a closed conformation, allowing phosphotyrosine to bind at the N-SH2 PBP with weaker (μM) affinity (*K*_*d2*_) (Fig. 3*B*) (50). The binding of phosphotyrosine to the N-SH2 PBP allosterically remodels the C-SH2 PBP to an intermediate affinity ($K_{d1}^{*}$). Our steady-state measurement of ITAM-ζ1-Y_2_P binding to an isolated wild-type tSH2 domain by isothermal calorimetric titration (ITC) revealed that potassium alters phosphotyrosine binding (*K*_*d2*_ and $K_{d1}^{*}$) in a concentration-dependent manner (Fig. S4*E*). To determine how elevated K^+^ alters ZAP-70 recruitment to TCR, we reevaluate the steady-state binding of ITAM-Y_2_P to C-SH2 (*K*_*d1*_) or N-SH2 (*K*_*d2*_) PBP with increasing KCl concentration (Fig. 3, *E* and *F* and Tables S4 and S5). We studied the binding of ITAM-ζ1-Y_2_P and C-SH2 PBP using a tSH2^R39A^ mutant, which impairs phosphate binding to the N-SH2 PBP. The strong (nM) ITAM-ζ1-Y_2_P and C-SH2 PBP interaction was probed by measuring the fluorescence polarization of an ITAM-ζ1-Y_2_P peptide labeled with AlexaFluor488 (Figs. 3*E*, S4*A* and Table S4). We probed the weaker (μM) ITAM-ζ1-Y_2_P and N-SH2 PBP interaction by ITC using a tSH2^R190A^ mutant, which impairs phosphate binding to the C-SH2 PBP (Figs. 3*F*, S4*D*, and Table S5). We selected the methods based on each technique’s sensitivity for measuring the respective binding strength. Our data suggest that increasing potassium concentration does not perturb the binding of ITAM-ζ1-Y_2_P and C-SH2 PBP (Figs. 3*E* and S4*A*). Unexpectedly, potassium perturbs the phosphotyrosine binding to N-SH2 PBP in a concentration-dependent manner (Fig. 3*F*). We observed that the N-SH2 PBP binds to the ITAM-ζ1-Y_2_P with a *K*_*d2*_ = 6.33 ± 0.34 μM in the presence of 150 mM NaCl (Fig. 3*F* and Table S3). We noticed that 5 mM KCl marginally affects the N-SH2 PBP and ITAM-ζ1-Y_2_P interaction (*K*_*d2*_ = 9.77 ± 0.61 μM) compared to NaCl (Fig. 3*F*). In contrast, increasing KCl concentration to 20 mM reduces the binding affinity of N-SH2 PBP for the ITAM-ζ1-Y_2_P by more than three times (*K*_*d2*_ = 20.93 ± 0.82 μM). Our biochemical measurements concur with cell-based studies, which explain why elevated [K^+^]_i_ prevents ZAP-70 clustering at the membrane (Fig. 3*H*). At higher K^+^ concentrations (>50 mM), we could not detect any interaction between the tSH2 domain and ITAM-ζ1-Y_2_P. Further analysis of structural stability (from ΔG_unfolding_) suggests that higher [K^+^] destabilizes the ligand-bound *holo*-tSH2 domain structure (compared to the *apo*-tSH2 domain) in a concentration-dependent manner (Figs. 3*G* and S4, *B* and *C*, and Table S6). The ΔG_unfolding_ determined at 150 mM KCl suggests that increasing K^+^ concentration favors unfolding of the holo tSH2 domain. We wonder why increasing potassium concentration preferentially destabilizes the tSH2 *holo*-state.

### Potassium slows down the structural transition of ZAP-70 to an active state in a concentration-dependent manner

**Figure 4:**
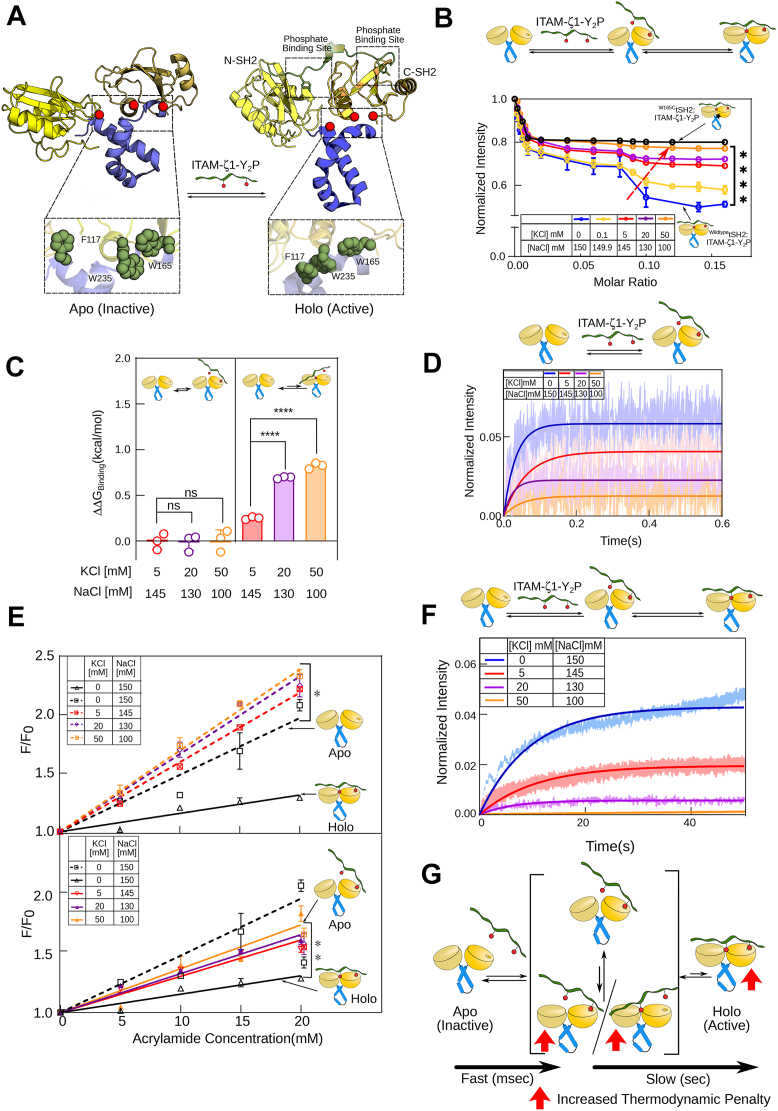
**A high potassium concentration increases the thermodynamic penalty for the interaction between the ITAM-Y_2_P and ZAP-70 tSH2 domains.***A*, the cartoon structure of the *apo* (PDB ID: 1M61) and *holo* (PDB ID: 2OQ1) ZAP-70 tSH2 domain. Inset highlights the conformation of the aromatic residues in the inactive and active states. *B*, a plot of changes in ZAP-70 tSH2 domain intrinsic tryptophan fluorescence against ligand to protein molar ratio. ITAM-ζ1-Y_2_P is titrated against the indicated construct of tSH2 domain in the presence of increasing KCl concentration. Each data point represents the mean ± SD from three independent experiments. The solid color lines are guiding lines. The ^W165C^tSH2 mutant found in SKG mice (51) is indicated in *black*. *C*, the change in ΔG_binding_ (ΔΔG_binding_) for the N-SH2 and C-SH2 phosphate binding pockets is plotted at the indicated KCl concentration. ΔΔG_binding_ is calculated with respect to the ΔG_binding_ obtained in the absence of KCl for the respective phosphate-binding pocket. *p* value from left to right: 0.4442, 0.9051, <0.0001, <0.0001. The tSH2 domain constructs described in Figure 3, *E* and *F* was used to probe ligand binding to the N-SH2 and C-SH2 PBP, respectively. *D* and *F*, the plots of time-dependent binding kinetics of the tSH2 domain (100 nM) and ITAM-ζ1-Y_2_P (15 μM) in the fast and slow time scales, respectively. *Solid lines* represent the single exponential fitting of the blank subtracted data. The area fill represents SD from three independent experiments. *E*, Stern-Volmer plot of normalized fluorescence intensity against increasing acrylamide concentration for the *apo* (*broken lines*) and *holo* (*solid lines*) tSH2 domain at the indicated KCl concentrations. The lines represent the fitting to a straight-line equation. The Stern-Volmer quenching constant (*K*_*sv*_) was determined from the slope of the fitted lines (see Fig. S6). *G*, a kinetic model of ZAP-70 tSH2 domain and ITAM-Y_2_P interaction (40). The *red up-arrows* indicate the steps where the thermodynamic penalty increases with increasing potassium concentration. *C* and *E*, a statistical analysis of two-tailed Students' t-tests was performed. Each data represents mean ± SD (ns = not significant; ∗*p* < 0.05; ∗∗*p* < 0.01; ∗∗∗*p* < 0.001; ∗∗∗∗*p* < 0.0001). All data were plotted using GraphPad PrismVer9.5.1. The schematics and icons were made using Inkscape Ver 1.4. See Figs. S4 and S5.

In the inactive state, the tSH2 domain of ZAP-70 adopts an open conformation where only the C-SH2 PBP is competent to bind the ITAM-Y_2_P (Fig. 4*A*) (40, 50, 52, 54). The final transition to the closed conformation of the *holo*-tSH2 domain requires the assembly of aromatic stacking interactions (the thermodynamic brake) that allosterically couple the two PBPs (Fig. 4*A*) (40). The tSH2 domain binds the ITAM-Y_2_P in multiple binding steps, producing a biphasic ligand-binding isotherm (Fig. 4*B*) (50). We probed the conformational rearrangement of the tSH2 domain during ITAM-ζ1-Y_2_P titration by measuring the changes in the intrinsic tryptophan fluorescence at steady-state (Fig. 4*B*) (50). We observed that increasing [K^+^] concentration by varying the NaCl to KCl ratio remodels the binding isotherm of ITAM-ζ1-Y_2_P and tSH2 domain to a hyperbolic (monophasic) pattern (Fig. 4*B*). At 50 mM KCl, the binding isotherm resembles the monophasic ligand binding reported previously for the ^W165C^tSH2 mutant (50). The W165 is a key residue in the allosteric network coupling the phosphotyrosine binding to the two PBPs (Fig. 4*A*). W165C mutation perturbs ligand binding to the N-SH2 PBP ($K_{d2}$), breaking the allosteric coupling between the C-SH2 and N-SH2 PBP (50). We speculate that K^+^ is weakening the ligand binding to the N-SH2 PBP, possibly by interfering with aromatic stacking through a cation-π interaction (56). It may be possible that, like the W165C mutation, higher [K^+^] prevents ITAM-Y_2_P binding by favouring an open conformation of the tSH2 domain. Therefore, we next investigated how potassium preferentially perturbs ligand binding to the N-SH2 PBP.

We recently demonstrated that the initial encounter complex between the ITAM-Y_2_P and C-SH2 domain forms through a fast-kinetic step, which transiently assembles the N-SH2 PBP (40). The final transition of the tSH2 domain to a closed conformation follows a slow kinetic step. Comparison of change in Gibbs’ free energy for ligand binding (${\Delta G}_{Binding}^{2}$) to the N-SH2 PBP shows that higher [K^+^] concentration imparts a greater thermodynamic penalty on ITAM-Y_2_P binding compared to Na^+^ (Fig. 4*C* and Table S5). For instance, compared to the ITAM-ζ1-Y_2_P binding measured at 5 mM KCl (${\Delta \Delta G}_{Binding}^{2}$ = 0.25 ± 0.012 kcal/mol), 20 mM KCl imposes a significant penalty (${\Delta \Delta G}_{Binding}^{2}$ = 0.69 ± 0.009 kcal/mol) on ligand binding to N-SH2 PBP (Fig. 4*C* and Table S5).

To determine if increasing [K^+^] affects the rate of structural rearrangement upon ligand binding, we measured the kinetics of the encounter complex formation and the final transition to the closed state. We probed the rate of structural rearrangement to the encounter complex and the final closed state by measuring the binding kinetics of ITAM-ζ1-Y_2_P to the tSH2 domain in the fast ($k_{obs}^{Fast}$) and slow ($k_{obs}^{Slow}$) kinetic steps. Using stopped-flow fluorescence spectroscopy, the tSH2 domain was mixed with excess ITAM-ζ1-Y_2_P dissolved in buffer with various NaCl and KCl concentrations (Fig. 4, *D* and *F* and Table S8). To probe the fast or slow kinetic step, changes in the intrinsic tryptophan fluorescence of the tSH2 domain after ligand mixing were recorded for 600 ms or 200 s, respectively. We observed that higher [K^+^] concentration does not alter the formation of the encounter complex (Figs. 4*D*, 3*E* and S5*C*). However, the reduced fluorescence intensity observed at higher [K^+^] concentrations indicates destabilization of the transiently formed NSH2 PBP (Fig. 4*D*). We observed that K^+^ significantly reduced the transition ($k_{obs}^{Slow}$) of the tSH2 domain to the final *holo*-state at higher concentrations (Figs. 4*F* and S5*D*). Suggesting that K^+^ disproportionately affects the structure of the closed conformation of the ligand-bound tSH2 domain.

**Figure 5:**
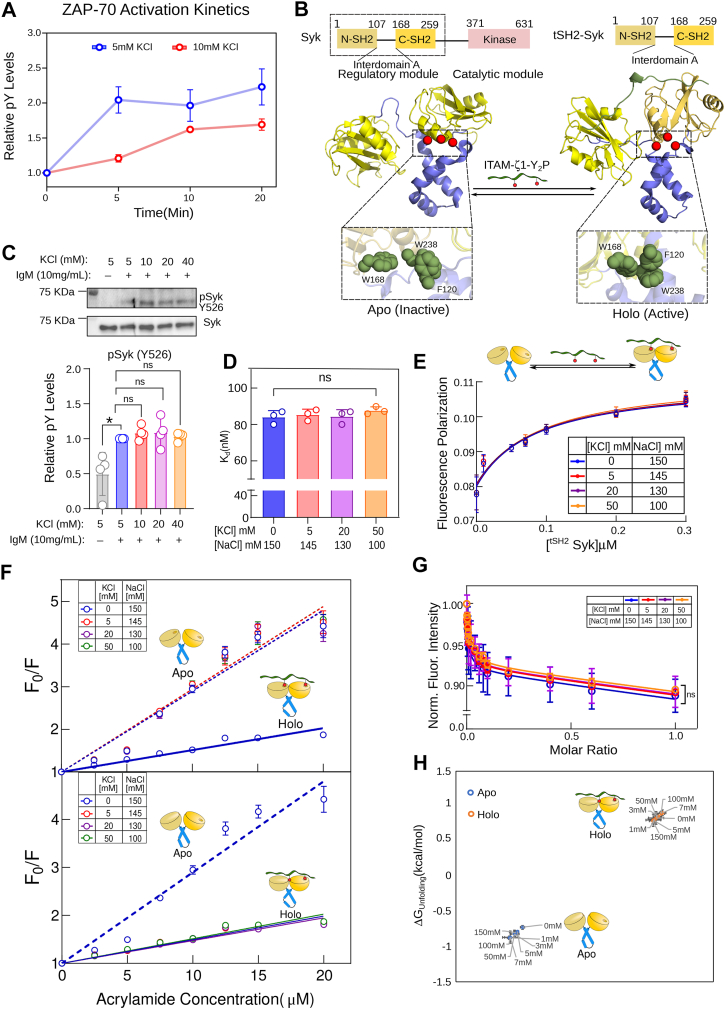
**A high potassium concentration does not alter the interaction between the Syk tSH2 domain and the ITAM-Y_2_P motif.***A*, the phosphorylation level of ZAP-70 Y493 upon TCR stimulation is plotted against time. The phosphorylation level is obtained from the densitometric analysis of immunoblot shown in Fig. S6*A*. Each data point represents the mean ± SD from three independent experiments. *B*, the *top panel* is the schematic representation of the domain architecture of Syk tyrosine kinase used in this study. The *bottom panel* is the structure of the tSH2 domain of Syk in the apo (PDB ID: 1A81) and holo (PDB ID: 4FL2) states. The conformation of the aromatic residues is shown in the inset. *C*, the *top panel* shows a representative immunoblot of Y526 phosphorylation and Syk expression in Ramos-RA1 B cell lines. The cells were activated with IgM in the presence of the indicated concentration of extracellular KCl. The *bottom panel* is the densitometric analysis of the immunoblots shown above. Bar graphs represent the fold changes in phospho-tyrosine levels. From left to right: *p* = 0.0170; *p* = 0.1529; *p* = 0.4141; *p* = 0.3127. *D*, the bar graph shows the *K*_*d*_ values for the Syk tSH2 domain and the ITAM-ζ1-Y_2_P interaction. The *K*_*d*_ represents the mean value from three independent ITC titrations of the indicated Syk construct and ITAM-ζ1-Y_2_P. *E*, the plot of fluorescence polarization as a function of Syk tSH2 domain concentration, determined from the titration of the tSH2 domain and ITAM-ζ1-Y2P tagged to Alexa Fluor488. Each data point is the mean ± SD from three independent experiments. The *colored solid lines* represent the fitting to the first-order binding. *F*, Stern-Volmer plot of normalized fluorescence intensity against increasing acrylamide concentration for the *apo* (*broken lines*) and *holo* (*solid lines*) tSH2 domain of Syk, at the indicated KCl concentrations. The lines represent the fitting to a straight-line equation. The Stern-Volmer quenching constant (*K*_*sv*_) was determined from the slope of the fitted lines. *G*, the plot of change in intrinsic tryptophan fluorescence in the tSH2 domain of Syk against the ligand-to-protein molar ratio at the indicated salt composition. *H*, ΔG_unfolding_ for the *apo* and *holo* tSH2 domain of Syk is plotted at the indicated KCl concentration. The ΔG_unfolding_ is derived from the thermal denaturation profile measured using Circular Dichroism (CD) spectroscopy (Fig. S6*G*). The data points were rearranged diagonally by plotting ΔG_unfolding_ on both axes. Each data point represents the mean ± SD from three independent experiments. *C* and *D*, a statistical analysis of two-tailed Students' t-tests was performed. Each data represents the mean ± SD from three independent experiments. (ns = not significant; ∗*p* < 0.05; ∗∗*p* < 0.01; ∗∗∗*p* < 0.001; ∗∗∗∗*p* < 0.0001). All data were plotted using GraphPad PrismVer9.5.1. The schematics and icons were made using Inkscape Ver 1.4. See Fig. S6.

To probe if elevating K^+^ would perturb the closed conformation of the tSH2 *holo*-state, we determined the Stern-Volmer quenching constant (*K*_*sv*_) by measuring acrylamide quenching of tryptophan fluorescence at increasing KCl to NaCl ratio. A higher *K*_*sv*_ value for the *apo* tSH2 domain (0.052 ± 0.004 μM^−1^) in buffer containing NaCl suggests that the tSH2 domain adopts an open conformation (Fig. 4*E* and Table S7). At the same time, shielding of acrylamide quenching of tryptophan fluorescence in the tSH2 *holo*-state (*K*_*sv*_ = 0.0164 ± 0.001 μM^−1^) indicates that the tSH2 domain adopts a closed conformation upon ligand binding, in the presence of NaCl. By increasing KCl concentration, the *K*_*sv*_ increases significantly for the *holo*-tSH2 domain (Figs. 4*E* and S5*B*). At 50 mM KCl, a *K*_*sv*_ value of 0.042 ± 0.002 μM^−1^ indicates that the tSH2 domain adopts a more open-like conformation even in the ligand-bound state. Our data suggest that higher [K^+^] does not perturb the formation of the encounter complex between ITAM-Y_2_P and the C-SH2 domain of ZAP-70 (Fig. 4*G*). However, the higher thermodynamic penalty imposed on the second ligand binding (at the N-SH2 PBP) significantly slows the assembly of the key aromatic-stacking interaction that allosterically couples structural rearrangement of the tSH2 domain to the active state. We anticipate that the impaired aromatic-stacking interaction, in turn, will prevent ZAP-70 activation by slowing its recruitment to the plasma membrane (Fig. 4*G*). Indeed, our studies using Jurkat E6.1 T cells show that increasing extracellular [K^+^]_e_ dampens the rate of ZAP-70 activation loop autophosphorylation (Fig. 5*A*). To summarize, our data indicate a central role of the aromatic stacking interaction (Fig. 4*A*) within the allosteric network of the tSH2 domain in sensing intracellular K^+^ dynamics.

### Syk tSH2 is insensitive to an increase in cellular potassium levels

To further validate the role of aromatic stacking interactions in the tSH2 domain of ZAP-70 in sensing the cellular K^+^ levels, we turned to Syk tyrosine kinase expressed in B cells. Syk is the paralogous kinase of ZAP-70 expressed in B cells that couples the B cell receptor (BCR) response to the downstream signaling (57). ZAP-70 and Syk share a similar structural architecture (Fig. 5*B*) and high sequence homology. Yet, the tSH2 domain of Syk binds to the ITAM-Y_2_P motifs in a hyperbolic manner, suggesting an altered ligand binding pattern compared to ZAP-70 (40, 58). ITAM-Y_2_P binding to the Syk tSH2 domain does not rely on the aromatic-stacking interaction (thermodynamic brake) (Fig. 5*B*) (40). Therefore, we speculate that the Syk in B cells will be active even in the presence of high extracellular [K^+^]_e_. Thus, we evaluate the effect of high [K^+^] concentration on the *in-cell* activation of the Syk kinase module and *in vitro* binding of the Syk tSH2 domain to ITAM-Y_2_P. We measured the *in-cell* activation of Syk by determining the activation loop Y526 phosphorylation in Ramos RA-1 cells after stimulating with IgM in the presence of elevated extracellular [K^+^]_e_. We observed no change in Syk activation loop autophosphorylation when Ramos RA-1 cells were activated at increasing [K^+^]_e_ concentrations (Fig. 5*C*). We evaluate the Syk tSH2 domain and ITAM-ζ1-Y_2_P interaction by ITC (Figs. 5*D*, S6*D*, and Table S10) and by fluorescence polarization experiment (Figs. 5*E*, S6*B*, and Table S9). As anticipated, high [K^+^] concentration did not disrupt the tSH2 and ITAM-ζ1-Y_2_P binding (Figs. 5, *D* and *E* and S6, *B*–*D*). We further compared the structure of the Syk (Fig. 5) and ZAP-70 (Figs. 3 and 4) tSH2 domain at various KCL concentrations from the Stern-Volmer quenching constant (*K*_*sv*_) (Figs. 5*F*, S6*E*, and Table S7), change in intrinsic tryptophan fluorescence upon ligand binding (Figs. 5*E*, S6*C*, and Table S9) and structural stability (from ΔG_unfolding_) (Figs. 5*H*, S6*F*, and Table S6). Our data revealed that, unlike the ZAP-70 tSH2 domain, elevated potassium neither perturbs the conformation nor disrupts the structural stability of the *apo* or the *holo* state of the Syk tSH2 domain. Increasing potassium concentration does not affect the *K*_*sv*_ values and the tryptophan fluorescence of the Syk tSH2 domain in the *holo* state. The differential response of ZAP-70 and Syk to K^+^ suggests that K^+^ does not influence the ligand binding at the PBP of the tSH2 domains. Instead, K^+^ interferes with the aromatic stacking in the ZAP-70 tSH2 domain, thereby uncoupling the structural transition of the encounter complex to the final active state (Fig. 4*G*).

### Proposed model of ionic suppression of ZAP-70-dependent early TCR signaling

**Figure 6:**
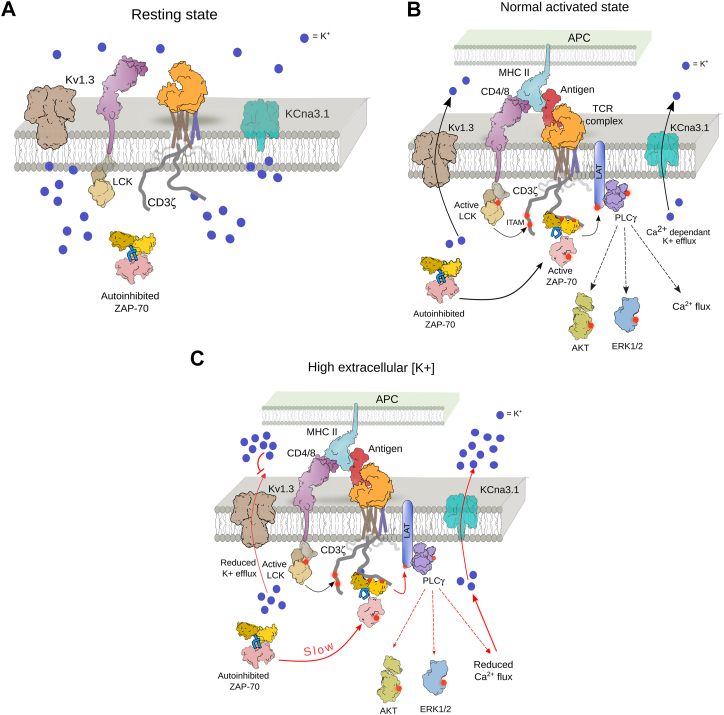
**A model of ionic suppression of ZAP-70-dependent early TCR signaling.***A–C*, the proposed model of ionic suppression of early TCR signaling. *Panel A*, represents the resting state of a T cell. Panels (*B* and *C*) stimulation of TCR in the absence and presence of elevated extracellular potassium, respectively. The binding of an antigen, presented by an APC, to the TCR initiates the sequential recruitment and activation of two tyrosine kinases, Lck and ZAP-70. Activation of ZAP-70 initiates subsequent downstream signaling. *Red arrows* indicate the steps perturbed by high extracellular potassium concentration. The schematics and icons were designed using Inkscape Ver1.4.

Our biochemical experiments suggest that high [K^+^]_i_ in the resting state interferes with the transition of the encounter complex to the final closed conformation of the tSH2 domain of ZAP-70 (Fig. 4*G*), preventing spontaneous T cell activation (Fig. 6*A*). Antigen binding to TCR brings down the intracellular potassium [K^+^]_i_ levels by inducing [K^+^]_i_ efflux (Figs. 1*C*, 6*B*, and S1*F*). Clustering of Kv1.3 channels at the TCR may reduce local intracellular [K^+^]_i_ levels (24, 26). Low intracellular [K^+^]_i_ levels, in turn, allow the recruitment of ZAP-70 to the phosphorylated CD3 chains in the TCR complex (Figs. 6*B* and 3*H*). High intracellular [K^+^]_i_ due to elevated extracellular [K^+^]_e_ mimicking TME or due to potassium channel blockers, prevents [K^+^]_i_ efflux upon antigen binding (Figs. 2*F*, 3*H*, 6*C*, and S1*F*). Thus, inhibiting the TCR signaling by altering the ZAP-70 recruitment to the ITAM-Y_2_P motif in the CD3-chains. It is possible that the disruption of the membrane potential, along with inhibition of ZAP-70-dependent downstream signaling, impairs Ca^2+^ influx from the ER and through CRAC channels (20, 59). Together, our data suggest that the intracellular [K^+^]_i_ dynamics are a key determinant of ZAP-70-dependent TCR response in quiescence or upon antigen binding (Fig. 6, *A*–*C*).

### Conclusion

Ion channels and ionic flux mediate diverse physiological functions of lymphocytes in health and disease. In the quiescent state, T cells maintain a high concentration of intracellular K^+^ (Figs. 1*C* and 6*A*) (1, 41). Early patch-clamp experiments supported the idea that antigen-binding to T cells induces K^+^ efflux, which sustains Ca^2+^ influx (Figs. 1, *C* and *D* and 5*B*) (23, 25). We observed that perturbing the gating properties of the K^+^ channels by increasing the extracellular [K^+^] concentration (60)or blocking potassium channels disrupts TCR-mediated K^+^ efflux and attenuates Ca^2+^ influx (Figs. 1, *C* and *D* and S1, *A* and *B*) (23, 45). Here, we presented a molecular mechanism of how intracellular K^+^ levels regulate TCR signaling.

Our data suggest that K^+^ interferes with the early step of TCR signaling. High intracellular K^+^ in the resting state or due to insufficient K^+^ efflux inhibits the recruitment of ZAP-70 to the TCR complex (Fig. 6*C*). Intriguingly, deleting cereblon (Crbn), an epigenetic repressor of Kcna3, in Crbn-deficient mice led to overexpression of Kv1.3 channels and ZAP-70, resulting in hyperactivation of CD4+ T cells (29). Suggesting that the expression of Kv1.3 channels and ZAP-70 is correlated. High intracellular K^+^ prevents ITAM-Y_2_P binding to the tSH2 domain of ZAP-70 by imparting a higher thermodynamic penalty on the structural transition to the final closed conformation (Fig. 4, *A* and *G*). The structural transition of the tSH2 domain is mediated by a network of allosteric interactions functioning as a thermodynamic brake. Central to the thermodynamic brake are aromatic stacking interactions, which are critical for ligand discrimination and sensing cellular K^+^ levels (Fig. 4*G*) (40). Our data suggest that K^+^ may prevent the assembly of a key aromatic stacking interaction, possibly by interacting with the π face of the aromatic rings (56) (Fig. 4*A*). Thus, increasing [K^+^] concentration alters the ZAP-70 function by imparting a higher thermodynamic penalty on the assembly of the aromatic stacking interaction. That, in turn, puts the brake on the structural transition of the tSH2 domain to the active state (Fig. 4*G*), slowing down the recruitment of ZAP-70 to the IS (Fig. 6, *A* and *C*). The non-functional thermodynamic brake (40) in the tSH2 domain of Syk renders the kinase insensitive to the cellular [K^+^] concentration (Fig. 5, *B*–*E*).

In an aqueous solution, K^+^ is preferred over Na^+^ to form a cation-π interaction with the aromatic residues in the allosteric network (56). Therefore, compared to Na^+^, K^+^ may impart a higher thermodynamic penalty on the ligand binding to the N-SH2 domains. Indeed, in all our biochemical experiments, we observed that increasing K^+^ concentration destabilises the interaction between ITAM-Y_2_P and the tSH2 domain. Together, our data explain why Na^+^ and K^+^ differentially regulate TCR signaling (1, 3, 4, 5).

In conclusion, we have demonstrated that intracellular potassium dynamics play a crucial role in regulating the physiological function of the TCR response. High intracellular potassium ensures low receptor activation in quiescent T cells by preventing ZAP-70 recruitment to the plasma membrane. We proposed that [K^+^]_i_ may influence the dwell time of ZAP-70 at the TCR by regulating the allosteric interaction between the tSH2 domain and the ITAM-Y_2_P motif in the CD3 chain (61). We conclude that intracellular potassium dynamics are an integral part of the kinetic proofreading for antigen discrimination by the TCR. The dependence of the ZAP-70 and ITAM-Y_2_P interaction on intracellular K^+^ levels suggests that cellular potassium dynamics may fine-tune the TCR response to self and non-self antigens.

## Experimental procedures

### Reagents and tools table

Table S1 in the supplementary information summarizes the list of antibodies and reagents.

### Methods and protocols

#### Cell culture

Jurkat E6.1 cells (ATCC # TIB-152), Jurkat P116 cells (ATCC # CRL-2676) and Ramos RA-1(ATCC # CRL-1596) were cultured in high glucose RPMI 1640 media (GIBCO #A10491–01) and HEK293T cells were cultured in DMEM media (GIBCO #11995049), supplemented with 10%FBS (GIBCO #10270-106) and 1% anti-anti (GIBCO#15240-062) at 37 °C and 5% CO_2_. Before activation, the cells were washed with PBS. 1 × 10^6^ cells (or 5 × 10^6^ as per experiment) were resuspended in serum-free RPMI media for 6 h at 37 °C. Following serum starvation, cells were resuspended in media containing various concentrations of KCl for an additional 4 h.

#### Constructs

The ZAP-70 tSH2 domain (residue number 1–256) was cloned into the pSKB2 vector, and full-length ZAP-70 was a gift from Prof. John Kuriyan, U.C. Berkeley. The point mutations were introduced into the tSH2 construct (R39A, R190A) using site-directed mutagenesis by PCR, as explained previously (50).

#### Intracellular potassium level estimation in Jurkat T-cells

The serum-starved Jurkat E6.1 T cells (at 5 × 10^6^ cells/ml) were loaded with PBFI-AM, probenecid, and Pluronic F127 by incubating for 3 hrs in RPMI-1640 media at 37 °C and 5% CO_2_ (42). Then the cells were washed twice in HBSS (composed of 0.4 mM Potassium Phosphate monobasic, 0.3 mM Sodium Phosphate dibasic, pH 7.4, 0.5 mM Magnesium Sulfate Heptahydrate, 0.5 mM Magnesium Chloride Hexahydrate, 4 mM Sodium Bicarbonate, 6 mM Glucose, 1 mM MgCl_2_) to remove the excess dye and resuspended in HBSS containing various proportions of KCl and NaCl. The fluorescence intensity of the cells was recorded by excitation at two wavelengths (λ_ex_) 340 and 380 nm, and the fluorescence intensity was recorded (λ_em_) at 505 nm with 5 s intervals. At the beginning of each experiment, the baseline (blank) was recorded for 5 min. The cells were then stimulated with OKT3 (1: 1000 dilution), and the change in PBFI fluorescence intensity was recorded for 30 min. In the control (blank) experiment, OKT3 was replaced by an equal amount of HBSS buffer. The intracellular KCl level was determined from the plot of normalized fluorescence intensity measured as a function of time. The PBFI-AM intensity was normalized from the ratio of fluorescence emission recorded at λ_ex_ 340 to λ_ex_ 380 nm.

#### Calcium signaling in Jurkat T-cells

The serum-starved Jurkat T-cells were treated with Fluo4 and incubated for 1 hour in HBSS (62, 63, 64, 65). The cells were washed twice in HBSS to remove the excess dye and resuspended in HBSS containing various proportions of KCl and NaCl. The fluorescence intensity of Fluo4 was measured with λ_ex_ of 495 nm and λ_em_ of 516 nm. At the beginning of each experiment, the baseline was recorded for 300 s, and then the cells were activated with OKT3 (1: 1000 dilution). A control (blank) experiment was performed for each condition where an equal volume of HBSS was added instead of OKT3. The calcium flux was estimated from the normalized Fluo4 fluorescence intensity plotted against time. The Fluo4 fluorescence intensity of the activated cells (F) was normalized with respect to the fluorescence intensity of the control (blank) experiment (F_0_).

#### Immunoblot analysis

The serum-starved Jurkat T-cells were stimulated with 1ug/ml human anti-CD3 mAb (OKT3) and 5ug/ml anti-CD28 mAb for 5 mins at 37 °C (or as indicated in the respective figures). While the Ramos RA-1 cells were activated with 10 μg/ml of IgM for 5 min. The phosphorylation is quenched by adding lysis buffer (final composition of 50 mM Tris pH 8, 150 mM NaCl, 1% NP40, 2 mM Na_3_VO_4_, 1 mM Benzamidine,10 mM NaF, 5 mM EDTA, 1x PhosSTOP (Roche #04906845001) (38). The cell lysate was placed on ice for 30 min, and cell debris was cleared by centrifuging at 13,000*g*. Protein samples were prepared by heating the supernatant with NuPAGE LDS sample buffer (4×) (Thermo #NP0007), resolved on SDS-PAGE, and blotted onto a PVDF membrane. The blotted membrane was blocked with 3% BSA dissolved in 1×TBS (20 mM Tris pH 7.6 and 150 NaCl) and 0.1% Tween-20 for 1 h at RT and then incubated with primary antibody at 4 °C overnight (dilution 1:1000). The unbound primary antibody was washed thrice with 1×TBST containing 0.1% tween-20, followed by incubation with a secondary antibody diluted in 1% BSA (dilution 1: 2500 for both anti-rabbit and anti-mouse HRP secondary antibody). The blot was washed three times with 1×TBST and two times with 1XTBS before developing with the Clarity Western ECL substrate kit (Bio-Rad #1705060). All images were acquired using the Bio-Rad Chemidoc system and analyzed using ImageJ. The details of the antibodies used and the dilutions are mentioned in Table S1. For potassium channel inhibition, cells were treated with 5 μM clofazimine (TCI #C2866) for 1 h before stimulation (45).

#### Flow Cytometric analysis to study phosphoprotein activation in Jurkat T-cells

The unstimulated and stimulated (for 5 min) Jurkat cells were immediately fixed in paraformaldehyde (final concentration of 4%) to quench the reaction. The cells were incubated at 25 °C for 30 min, and then washed once with FACS buffer (2% FBS, 1 mM EDTA in PBS buffer), and resuspended in 100% ice-cold methanol for 30 min at 4 °C to permeabilize. The cells were rehydrated with FACS buffer for 1 hour on ice and stained with specific anti-phosphotyrosine- or anti-protein antibodies for 1 hour at room temperature. The cells were washed twice in FACS buffer and then stained with a secondary antibody conjugated to Alexa Fluor-488 or AlexaFluor-647 for 45 min at room temperature. The cells were washed twice in FACS buffer and analyzed using BD LSRFortessa flow cytometer (BD Biosciences) (38, 66).

#### Stable cell line preparation by lentiviral transduction

Human full-length ZAP-70 fused to EGFP at the C-terminal was cloned into second-generation lentivirus vector pLVX M.Puro (Addgene #125839). To make the final lentivirus carrying the ZAP-70-EGFP, the cloned pLVX plasmid is cotransfected along with packaging vector psPAX2 (Addgene #12260) and envelope vector pMD2.G (Addgene #12259) (in 4:3:1 ratio) into HEK293T cells (67). The supernatants containing the virus were collected 48 h post-transfection, filtered using 0.45 μm filters, and stored at −80 °C. For stable cell line preparation, Jurkat P116 cells were activated using OKT3/anti- CD28 mAb (1 μg/ml and 5 μg/ml) for 10 min and then transduced with the lentiviral titer and incubated for 48 hours at 37 °C and 5% CO_2_. Jurkat P116 cells expressing ZAP-70-EGFP were sorted using BD FACSAria III and cultured in fresh complete RPMI media (GIBCO A1049101) with 15% FBS (GIBCO 10270-106) and 1% anti-anti (GIBCO 15240062).

#### Live cell imaging using TIRF microscopy

Jurkat P116 cells stably expressing ZAP-70-EGFP were incubated for 3 h in RPMI media containing 5 mM KCl or 20 mM KCl. For imaging, the cells adhered to a glass coverslip coated with human fibronectin (Sigma-Aldrich F2006) for 1 h at 37 °C and in 5% CO_2_. The cells were then imaged using TIRF microscopy with a time-lapse of 5 s/frame, utilizing an inverted microscope (Olympus IX-83, Olympus, Japan) equipped with a 100X 1.49 NA oil immersion TIRF objective (PlanApi, Olympus). The imaging setup included an s-CMOS camera (ORCA Flash 4.0, Hamamatsu, Japan) and a 488 nm laser source. Images were acquired with an exposure time of 200 ms, achieving a penetration depth of approximately 70 nm. The live cells were activated using 1 μg/ml anti-human-CD3 mAb (OKT3) (BD Biosciences, 567,107), and time-lapse images of the activated cells were captured.

#### Image analysis

Image analysis was performed using Fiji Ver1.5. A Gaussian blur operation was performed for cluster analysis on the TIRF images. The blurred image was subtracted from the raw image, thus enhancing the local contrast. The thresholding was performed using the appropriate threshold, resulting in a binary image from which five randomly appearing clusters of 10 pixels (1 pixel = 0.65 μm) or larger size were chosen from the cells. The intensity of the chosen clusters was captured over time. The rate of ZAP-70 recruitment to the plasma membrane was determined from the plot of change in EGFP intensity over time. The total cluster number (≥10-pixel size) from the individual cell was determined and analyzed using GraphPad PrismVer9.5.1.

#### Expression and purification of tandem SH2 domain of ZAP-70

The tSH2 domains of ZAP-70 and the mutants were overexpressed in *E.coli*-BL21(DE3) cells. The culture was induced with 1 mM IPTG overnight at 18 °C. Cells were lysed by sonication in lysis buffer containing 50 mM Tris, pH 8, 200 mM NaCl, 20 mM imidazole, 5 mM β-mercaptoethanol, and 5% glycerol. The tSH2 domain was purified as described previously (40, 50). Briefly, the clear cell lysate was passed through a Ni-NTA affinity column and eluted with an imidazole-containing elution buffer composed of 50 mM Tris, pH 8, 200 mM NaCl, 500 mM imidazole, 5 mM β-mercaptoethanol, 5% glycerol. The eluate from the Ni-NTA column was further purified using a Q-column followed by gel-filtration chromatography. The purified tSH2 domain was concentrated and stored in 20 mM Tris, pH 8, 150 mM NaCl, 5 mM β-mercaptoethanol, and 5% Glycerol at −80°C.

#### Expression and purification of the tandem SH2 domain of Syk

The tSH2 domain of Syk is expressed as GST fusion in *E.coli*-BL21 (DE3) cells as described previously (40). The cells were lysed by sonication in 1 × PBS pH7.4. The cell lysate was passed through the GST affinity column and eluted with L-glutathione-containing elution buffer composed of 50 mM Tris, pH 8.2, 10 mM Reduced Glutathione, and 10% Glycerol. The eluted protein was digested overnight using Precision Protease and loaded on a GST column again to remove the cleaved GST tag. The protein was further purified using gel-filtration chromatography. The purified protein was concentrated and stored in 50 mM Tris, pH 8, 150 mM NaCl, 5 mM β-mercaptoethanol, and 10% Glycerol at −80 °C.

#### Fluorescence polarization experiments

The interaction between the tSH2 domain and ITAM-ζ1-Y_2_P was quantitatively determined from the steady-state fluorescence anisotropy experiment. The indicated constructs of the tSH2 domain of ZAP-70 were titrated against the 25 nM ITAM-ζ1-Y_2_P labeled with AlexaFluor 488. Both the labeled peptide and the tSH2 domain were dissolved in 20 mM Tris, pH 8, 150 mM NaCl, 5 mM β-mercaptoethanol, 5% Glycerol. The fluorescence polarization was recorded using a Hitachi (model F-4500) fluorometer equipped with a polarizer. To test the effect of increasing potassium concentration on the ITAM-ζ1-Y_2_P and tSH2 binding, the titration was repeated with various ratios of NaCl and KCl-containing buffers, keeping the total salt concentration at 150 mM. The dissociation constant was determined by fitting the curve to the following equation implemented in GraphPad PRISM: $Y=Y_{0}+\left[\frac{B_{\max}\times X}{K_{d}+X}\right]$. Where *Y* is the anisotropy measured in the presence of protein, *Y*_*0*_ is the anisotropy measured for the free ITAM-ζ1-Y_2_P peptide, X is the concentration of the protein, B_max_ is the maximum value of anisotropy measured, and *K*_*d*_ is the dissociation constant.

#### Thermal unfolding assay using circular dichroism

The melting temperature (T_m_) of the *apo* or *holo* tSH2 domain of ZAP-70 in various KCl concentrations was determined from the thermal unfolding of the proteins measured using a circular dichroism (CD) spectrophotometer (Jasco-J810 spectrophotometer). For the CD experiment, the protein and the peptide were dissolved in 20 mM phosphate buffer pH 7.4 and the various concentrations of NaCl or KCl, keeping the total salt concentration at 150 mM. Two CD spectra of the tSH2 domain were recorded for each experimental condition, one in the *apo* state and one in the *holo* state (1:1 complex of tSH2: ITAM-ζ1-Y_2_P). For each data set, the spectrum was scanned between 300 to 200 nm at a temperature ranging from 20 to 60 °C with an increment of 4 °C. The ellipticity data were converted into molar ellipticity using the following equation (68): $\left[\theta \right]=\frac{m_{0}\times M}{\left(10\times L\times C\right)}$.

The change in Gibbs free energy of unfolding (Δ*G*_unfolding_) for the *apo* or *holo* tSH2 domain was derived assuming the simplest two-step unfolding model (68, 69). The fraction unfolded [U] protein at a given temperature was derived using: $\left[U\right]=\frac{\left(\theta_{T}-\theta_{F}\right)}{\left(\theta_{U}-\theta_{F}\right)}$. Where $\theta_{F}$ and $\theta_{U}$ are the molar ellipticity of the fully folded and unfolded state at 222 nm, $\theta_{T}$ is the molar ellipticity at a given temperature. The equilibrium constant, $K_{unfolding}$, was calculated using the following equation: $K_{unfolding}=\frac{\left[U\right]}{\left(1-\left[U\right]\right)}$. The ΔG_unfolding_ was derived from: ${\Delta G}_{unfolding}=-RTln\left(K_{unfolding}\right)$, where R = 1.98 × 10^−3^ kcal mol^−1^ and T = 317K (44 °C). Related to Figure 5*G*.

#### Isothermal titration calorimetry

Malvern PEAQ-ITC was used to determine the affinity of the N-SH2 phosphate binding pocket for the ITAM-ζ1-Y2P peptide at various KCl concentrations. 20 μM of ZAP-70 ^R190A^tSH2 domain was titrated with increasing ITAM-ζ1-Y_2_P concentrations in glycerol-free buffer composed of 20 mM HEPES, pH 8.2, 5 mM β-mercaptoethanol and indicated proportion of NaCl and KCl. A stock concentration of 300 μM of ITAM- ζ1-Y2P was loaded in the syringe. Each experiment consisted of nineteen injections of 2 μl each and a delay of 180 s between the injections. The protein solution was stirred at 300 rpm during the titration at 20 °C. The *K*_*d*_, ΔH, and ΔS were obtained by fitting observed heat exchanged from the titration to a one-site ligand-binding model implemented in the MicroCal PEAQ-ITC Analysis Software v1.41.

#### Steady-state fluorescence experiments

The interaction between the tSH2 domain and ITAM-ζ1-Y_2_P peptide in the steady-state was determined from the change in intrinsic tryptophan fluorescence. The tryptophan fluorescence was measured at λ_ex_ 295 nm, and the emission spectrum (λ_em_) was scanned between 300 nm to 400 nm at 25 °C. The fluorescence spectra were recorded using a Horiba Duetta spectrophotometer. For each titration, 1 μM tSH2 domain was titrated with various concentrations of ITAM-ζ1-Y_2_P peptide. The peptide and the protein were dissolved in 20 mM Tris pH 8, 5% glycerol, 5 mM β-mercaptoethanol, and different concentrations of NaCl or KCl, keeping the total salt concentration at 150 mM. The normalized fluorescence intensity (F_0_/F) at 340 nm (λ_em_) was plotted against the ligand-to-protein molar ratio. Where F_0_ and F are the intrinsic tryptophan fluorescence in the absence and presence of ligands, respectively.

#### Acrylamide quenching experiments

The structure of the tSH2 domain in the open (*apo*) or closed (*holo*) conformation was evaluated from the quenching of intrinsic tryptophan fluorescence by acrylamide at the indicated salt concentration. The tryptophan fluorescence for the *apo* or *holo* tSH2 domain was measured by titrating an increasing acrylamide concentration. The fluorescence emission was scanned between 300 and 400 nm. The fluorescence intensity at 340 nm was plotted against the respective acrylamide concentration. The Stern-Volmer quenching constant (*K*_*sv*_) was determined from the linear fitting of the data: $\frac{F_{0}}{F}=1\;+\;(K_{sv}\;\times \;\;X)$ (50, 70). Where F_0_ and F are the fluorescence in the absence and presence of acrylamide, respectively. X is the concentration of the acrylamide.

#### Pre-steady state kinetics of ITAM-ζ1-Y2P and ZAP-70 tSH2 interaction using stopped-flow fluorescence spectroscopy

The binding kinetics of the ZAP-70 tSH2 domain and ITAM-ζ1-Y_2_P peptides were measured from the change in intrinsic tryptophan fluorescence of the tSH2 domain at 10 °C. The tryptophan fluorescence was recorded with an SFM2000 BioLogic spectrophotometer fitted with a stopped-flow system. The protein and the peptide were dissolved in a buffer containing 20 mM Tris (pH 8.0), 5% glycerol, 5 mM β-mercaptoethanol, and different concentrations of NaCl or KCl, as indicated in Figure 6, *D* and *F*. Each kinetic experiment was carried out by mixing 100 nM of tSH2 domain with 15 μM of ITAM-ζ1-Y_2_P and recorded over 200s (for slow kinetics) or one second (for fast kinetics), respectively. A blank dataset was recorded for each sample by measuring the change in intrinsic tryptophan fluorescence of tSH2 domains upon mixing with buffer. Each data set was normalized against the maximum intensity observed for the sample at t = 0. The normalized data set was further corrected by blank subtraction. The observed rate constant ($k_{obs}^{fast}$ or $k_{obs}^{slow}$) was derived by fitting to an association kinetics equation using PRISM: $Y=Y_{0}+\left(Y_{\max}-Y_{0}\right)(1-e^{-k_{obs}t}$). where *Y*_*0*_ is the intensity at t = 0, Y_max_ is the maximum intensity, *k*_*obs*_ is the rate constant.

#### Statistics and reproducibility

Statistical analyses were performed with GraphPad Prism 9 using an unpaired two-tailed Student’s *t* test. No statistical method was used to determine the sample size in advance. No data were excluded from the analyses. All data were presented as mean ± SD from at least three biologically independent experiments. *p* < 0.05 was considered statistically significant.

## Data availability

All the relevant data are contained within this article and in the Supplemental Materials.

## Supporting information

This article contains supporting information.

## Conflict of interests

The authors declare that they have no conflict of interest with the contents of this article.
