# Supplementary material for: Potassium suppresses allosteric activation of ZAP-70-dependent T cell receptor signaling
Source: J Biol Chem. 2026 Apr 28;302(6):113083. doi: 10.1016/j.jbc.2026.113083 (PMC13235457; doi:10.1016/j.jbc.2026.113083)
Supplement: Supporting information [file mmc1.pdf]

Potassium suppresses allosteric activation of ZAP-70-dependent T Cell Receptor signalling

Swarnendu Roy[a]#, Soumee SenGupta[a]#, Kaustav Gangopadhyay[a], Sudipta Majumder[a], Jibitesh Das[a], Anushka Sinha[a], Prosad Kumar Das[a], Bidisha Sinha[a], and Rahul Das[a],[b]*

[a] Department of Biological Sciences, Indian Institute of Science Education and Research Kolkata, Mohanpur campus, Mohanpur-741246, India

[b] Centre for Advanced Functional Materials, Indian Institute of Science Education and Research Kolkata, Mohanpur campus, Mohanpur-741246, India

# Authors contributed equally

Supplemental Materials

*Corresponding authors

Rahul Das: rahul.das@iiserkol.ac.in

Table S1: **List of Antibodies and Reagents**

Antibody	Details	Source	Catalog Number	Lot Number	Dilution
Anti- Human CD3 Clone: OKT3	Purified Mouse Anti-Human	BD Pharmagen	567107	3059362	1:1000 (Stimulation)
Human CD28 Clone:37407	Mouse Monoclonal	R&D Systems	MAB342	AEG1124021	1:200 (Co-stimulation)
Anti-Human IgM	Goat Polyclonal	Sigma-Aldrich	12386	118M4782V	1:100 (Stimulation)
Human LCK Clone:693010	Mouse Monoclonal	R&D Systems	MAB37041	CEWU012407B	1:1000 (Immunoblotting)
Anti-Human phospho LCK (Y394) Clone:755103	Mouse Monoclonal	R&D Systems	MAB7500	CGED0223041	1:1000 (Immunoblotting)
Anti-CD3ζ	Rabbit Monoclonal	Cell Signaling Technology	88083	1	1:1000 (Immunoblotting)
Anti-phospho CD3ζ (Y142)	Rabbit Monoclonal	Cell Signaling Technology	67748	1	1:1000 (Immunoblotting)
Anti-ZAP-70 (99F2)	Rabbit Monoclonal	Cell Signaling Technology	2705S	12	1:1000 (Immunoblotting)
Anti-phospho ZAP-70 (Y493)	Rabbit Monoclonal	Cell Signaling Technology	2704S	10	1:1000 (Immunoblotting)
Anti-Syk (D3Z1E)	Rabbit Monoclonal	Cell Signaling Technology	13198S	9	1:1000 (Immunoblotting)
Anti-LAT	Rabbit Polyclonal	MyBioSource	MBS2091781	A202003	1:1000 (Immunoblotting)
Anti-phospho LAT (Y191)	Rabbit Monoclonal	MyBioSource	MBS82203	CP1D10A	1:1000 (Immunoblotting)
Anti-GAPDH	Rabbit Monoclonal	BioBharati Life Sciences	BB-AB0060	011501	1:1000 (Immunoblotting)
Anti-PLCγ	Rabbit Monoclonal	Cell Signaling Technology	5690S	7	1:1000 (Immunoblotting)
Anti-phospho PLCγ (Y783)	Rabbit Monoclonal	Cell Signaling Technology	14008S	4	1:1000 (Immunoblotting)

Anti-phospho p44/42 MAPK (T202/Y204) (D13.14.4E)	Rabbit Monoclonal	Cell Signaling Technology	4370S	28	1:200 (Flow cytometry)
Anti-phospho AKT (S473) (193812)	Rabbit Monoclonal	Cell Signaling Technology	4058T	30	1:200 (Flow cytometry)
Anti-rabbit IgG Secondary (HRP)	Goat anti-rabbit	Abcam	Ab6717	GR267728-27	1:2500 (Immunoblotting)
Anti-mouse IgG Secondary (HRP)	Goat anti-mouse	Cell Signalling Technology	7076S	33	1:2500 (Immunoblotting)
Anti- total Phosphotyrosine	Rabbit monoclonal	Abcam	AB179530	1000218-13	1:1000 (Immunoblotting)
Anti-rabbit IgG (H+L) Alexa Fluor 488	Goat anti-Rabbit	Invitrogen	A11008	2897813	1:100 (Flow cytometry)
Anti-rabbit IgG (H+L) Alexa Fluor 647	Goat anti-Rabbit	Invitrogen	A31573	2752586	1:100 (Flow cytometry)

List of Chemicals and peptides:

Chemicals / Peptides	Source	Catalog Number
PBFI, AM, Cell Permeant-Special Packaging	Thermo Fischer Scientific	P1265MP
Fluo-4 NW Calcium Assay Kit	Thermo Fischer Scientific	F36206
Clofazimine	TCI	C2866
ITAM-ζ1-Y_2P peptide	S Biochem	NA
AlexaFluor 488 tagged ITAM-ζ1-Y_2P peptide	GenPro Biotech	NA
PhosSTOP	Roche	04906845001
Clarity ™ Western ECL substrate kit	Bio-Rad	1705060
NuPAGE LDS sample buffer (4X)	Thermo Fischer Scientific	NP0007

Table S2: Amplitude of calcium signaling measured at t = 540 sec.

[KCl]mM	[NaCl]mM	Amplitude*(AU)
5	145	2.75±0.28
10	140	2.2±0.02
20	130	1.48±0.08
50	100	1.47±0.187
Clofazimine		
5	145	1.13±0.005

* The amplitude values represent the mean of three separate experiments and the associated standard deviation

Table S3: Rate of decay of PBFI intensity and the intensity of PBFI measured at t = 1200 sec under various salt concentrations and clofazimine treatment.

[KCl]mM	[NaCl]mM	Rate of Decay (AU/s)*	Relative PBFI ratio *
5	145	62.3±3.78 X 10^{-5}	0.56±0.07
10	140	44.7±8.26 X 10^{-5}	0.68±0.03
20	130	24.3±8.8 X 10^{-5}	0.73±0.11
50	100	34.7±3.68 X 10^{-5}	0.77±0.03
Clofazimine			
5	145	1.53±0.55 X 10^{-5}	0.98±0.008

* The values represent the mean of three separate experiments measured at 1200 sec and the associated standard deviation.

Table S4: Binding parameters of ITAM-ζ1-Y_2P and the C-SH2 phosphate binding pocket determined from fluorescence polarization experiment.

[KCl]mM	[NaCl]mM	K_{d1} (nM)*	$\Delta G^{1}_{Binding}$ (kcal/mol)*	$\Delta\Delta G^{1}_{Binding}$ (kcal/mol)*
0	150	4.52±0.93	-11.16±0.12	--
5	145	4.44±0.69	-11.17±0.1	-0.006±0.071
20	130	4.34±0.42	-11.17±0.06	-0.015±0.074
50	100	4.53±0.66	-11.15±0.087	0.006±0.094

* The values represent the mean of three separate experiments and the associated standard deviation

Table S5: Binding parameters of ITAM-ζ1-Y_2P and the N-SH2 phosphate binding pocket determined from isothermal titration calorimetry experiment.

[KCl]mM	[NaCl] mM	K_{d2} (µM)*	$\Delta G^{2}_{Binding}$ (kcal/mol)*	-TΔS (kcal/mol)*	ΔH (kcal/mol)*	N	$\Delta\Delta G^{2}_{Binding}$ (kcal/mol)*
0	150	6.33±0.34	-6.95±0.031	5.6±0.1	-11.5±0.17	0.9	--
5	145	9.77±0.61	-6.69±0.037	4.75±0.12	-10.4±0.21	0.9	0.25±0.012
20	130	20.93±0.82	-6.25±0.023	3.04±0.08	-9.35±0.196	0.9	0.69±0.009
50	100	26.17±0.85	-6.12±0.02	2.64±0.14	-8.77±0.11	0.9	0.82±0.027

* The values represent the mean of three separate experiments and the associated standard deviation

Table S6: Change in Gibbs free energy for unfolding ($\Delta G_{unfolding}$) measured from the thermal denaturation of *apo* and *holo* tSH2 domain of ZAP-70 and Syk using CD spectroscopy**.**

**ZAP-70 tSH2**			
Apo			
[KCl] mM	[NaCl] mM	Melting Temperature T_m(°C)	$\Delta G_{unfolding}$ (kcal/mol)
0	150	39.13±1.375	-0.405±0.135
1	149	40.02±0.57	-0.431±0.105
3	147	40.24±1.065	-0.862±0.175
7	143	40.58±0.565	-0.550±0.068
10	140	38.04±0.53	-0.528±0.237
50	100	37.54±1.51	-0.407±0.129
100	50	37.29±0.585	-0.963±0.099
150	0	38.27±0.645	-0.781±0.050
Holo			

[KCl] mM	[NaCl] mM	Melting Temperature T_m(°C)	$\Delta G_{unfolding}$ (kcal/mol)
0	150	47.79±0.27	1.083±0.033
1	149	46.88±0.295	0.880±0.012
3	147	46.45±0.3	0.770±0.071
7	143	45.69±0.375	0.736±0.005
10	140	45.67±0.63	0.651±0.021
50	100	45.61±0.2	0.570±0.035
100	50	45.33±0.195	0.493±0.064
150	0	44.37±0.28	0.434±0.087
**Syk tSH2**			
Apo			
[KCl] mM	[NaCl] mM	Melting Temperature T_m(°C)	$\Delta G_{unfolding}$ (kcal/mol)
0	150	45.1±0.1	0.118±0.036
1	149	44.5±0.1	0.040±0.019
3	147	44.73±0.15	0.008±0.056
7	143	44.87±0.058	0.121±0.016
50	100	44.53±0.252	0.014±0.057
100	50	44.73±0.208	0.113±0.005
150	0	44.53±0.351	0.105±0.086
Holo			
[KCl] mM	[NaCl] mM	Melting Temperature T_m(°C)	$\Delta G_{unfolding}$ (kcal/mol)
0	150	51.3±0.35	0.935±0.11
1	149	51.26±0.305	0.79±0.12
3	147	51.43±0.153	0.89±0.13
7	143	51.2±0.35	0.995±0.083
50	100	51.63±0.153	0.965±0.147
100	50	51.37±0.153	0.921±0.07
150	0	51.57±0.153	0.94±0.035

* $\Delta G_{unfolding}$ is calculated at T= 317K (44°C).

Table S7: Stern-Volmer quenching constant (K_{sv}) for the *apo* and *holo* tSH2 domain of ZAP-70 and Syk at indicated salt concentration.

**ZAP-70 tSH2**		
Apo		
**[KCl]mM**	**[NaCl]mM**	**K_{sv} (μM^{-1})***
0	150	0.052±0.004
5	145	0.062±0.004
20	130	0.066±0.004
50	100	0.068±0.001
Holo		
**[KCl]mM**	**[NaCl]mM**	**K_{sv} (μM^{-1})***
0	150	0.0164±0.001
5	145	0.0278±0.001
20	130	0.03±0.001
50	100	0.042±0.002
**Syk tSH2**		
Apo		
**[KCl]mM**	**[NaCl]mM**	**K_{sv} (μM^{-1})***
0	150	0.047±0.0002
5	145	0.043±0.0015
20	130	0.044±0.0009
50	100	0.045±0.0019
Holo		
**[KCl]mM**	**[NaCl]mM**	K_{sv} (μM^{-1})*
0	150	0.198±0.0083
5	145	0.202±0.0081
20	130	0.196±0.0067
50	100	0.204±0.0067

* The values represent the mean of three separate experiments and the associated standard deviation

Table S8: Observed rate (k_{obs}) for the ZAP-70 tSH2 domain and ITAM-ζ1-Y_2P binding measured at indicated salt concentration.

Fast Binding (k_{obs}^{Fast})		
**[KCl]mM**	**[NaCl]mM**	**k_{obs} (s^{-1})***
0	150	21.74±2.52
5	145	25.68±2.79
20	130	25.07±4.61
50	100	Not Determined
Slow Binding (k_{obs}^{Slow})		
0	150	0.23±0.007
5	145	0.132±0.013
20	130	0.1±0.0003
50	100	Not Determined

* The values represent the mean of three separate experiments and the associated standard deviation

Table S9: Dissociation constant (K_d) determined from the titration of ITAM-ζ1-Y_2P and the tSH2 domain of Syk by measuring the changes in intrinsic tryptophan fluorescence and fluorescence polarization.

[KCl] mM	[NaCl] mM	K_d (nM)* (Intrinsic Tryptophan Fluorescence Titration)	K_d (nM)* (Fluorescence Polarization)
0	150	97.54±18.5	85.49±13.49
5	145	86±10.4	85.49±11.74
20	130	84.3±12.1	81.1±7.92
50	100	82.67±12.01	84.89±11.05

* The values represent the mean of three separate experiments and the associated standard deviation

Table S10: Binding parameters of ITAM-ζ1-Y_2P and the Syk tSH2 domain determined from isothermal titration calorimetry experiment.

[KCl]mM	[NaCl] mM	K_d (µM)*	$\Delta G_{Binding}$ (kcal/mol)*	-TΔS (kcal/mol)*	ΔH (kcal/mol)*	N
0	150	84±2.94	-9.49±0.021	5.87±0.429	-15.37±0.45	0.8
5	145	85.33±2.49	-9.7±0.136	4.84±0.143	-14.54±0.18	0.87
20	130	84.33±3.09	-9.57±0.13	4.16±0.06	-13.73±0.17	0.8
50	100	87.67±1.67	-9.57±0.06	3.93±0.153	-13.47±0.21	0.8

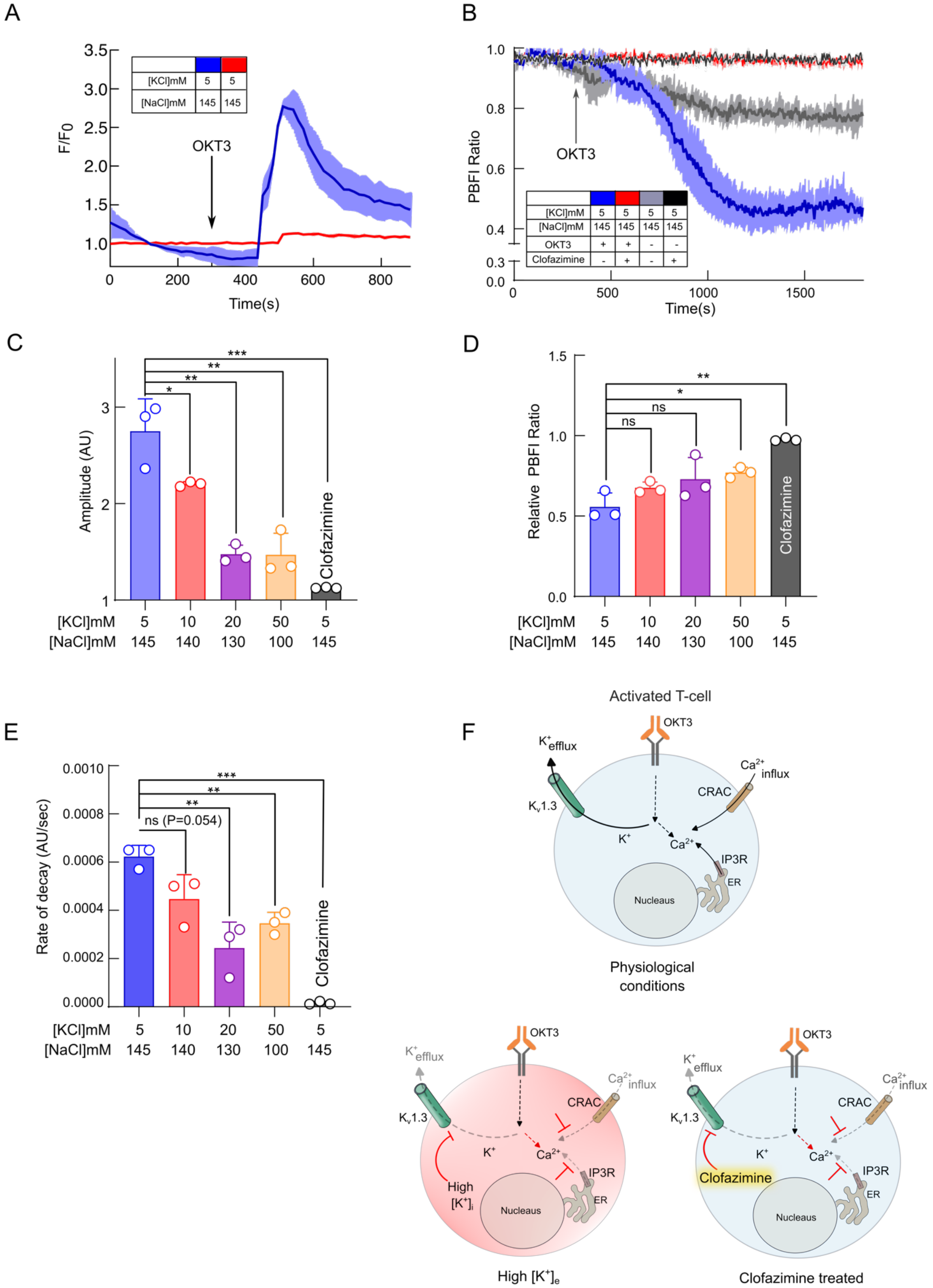
A
3.5
3.0
2.5
2.0
1.5
1.0
F/F0
[KCl]mM 5 5
[NaCl]mM 145 145
OKT3
0
200
400
600
800
Time(s)
B
1.0
0.8
0.6
0.4
0.3
0.0
PBFI Ratio
OKT3
[KCl]mM 5 5 5 5
[NaCl]mM 145 145 145 145
OKT3 + + - -
Clofazimine - + - +
0
500
1000
1500
Time(s)
C
Amplitude (AU)
3
2
1
***
**
**
*
Clofazimine
[KCl]mM 5 10 20 50 5
[NaCl]mM 145 140 130 100 145
D
1.5
1.0
0.5
0.0
Relative PBFI Ratio
**
*
ns
ns
Clofazimine
[KCl]mM 5 10 20 50 5
[NaCl]mM 145 140 130 100 145
E
Rate of decay (AU/sec)
0.0010
0.0008
0.0006
0.0004
0.0002
0.0000
***
**
**
ns (P=0.054)
Clofazimine
[KCl]mM 5 10 20 50 5
[NaCl]mM 145 140 130 100 145
F
Activated T-cell
OKT3
K+ efflux
Ca2+ influx
CRAC
Kv1.3
K+
Ca2+
IP3R
ER
Nucleaus
Physiological conditions
OKT3
K+ efflux
Ca2+ influx
CRAC
Kv1.3
K+
Ca2+
IP3R
High [K+]i
ER
Nucleaus
High [K+]e
OKT3
K+ efflux
Ca2+ influx
CRAC
Kv1.3
K+
Ca2+
Clofazimine
IP3R
ER
Nucleaus
Clofazimine treated

Figure S1: Potassium channel inhibitor, clofazimine, prevents TCR-dependent potassium efflux and attenuates calcium signaling.

A) Calcium flux was measured from the plot of normalized Fluo4 fluorescence intensity as a function of time (seconds) on stimulation of Jurkat E6.1 T-cells in the absence (blue solid line) and presence of clofazimine (red solid line). The T-cells were stimulated with anti-CD3 antibody (OKT3), at the indicated time. The fluorescence intensity for the activated (F) T-cells is normalized against the inactive (F_o) cells. The solid lines represent the mean from three independent experiments, and the area fill denotes the standard deviation.

B) The potassium efflux is measured from the plot of PBFI fluorescence intensity as a function of time in the Jurkat E6.1 T-cells in the presence (red) and absence (blue) of clofazimine. The T-cells were activated with an anti-CD3 antibody (OKT3), at the indicated time. The fluorescence intensity from uninduced cells is represented as solid black and gray lines. The solid lines represent the mean from three independent experiments, and the area fill denotes the standard deviation.

C) The bar graph represents the amplitude of calcium signaling in activated Jurkat E6.1 T-cells under the indicated experimental conditions. From left to right P = 0.0488, P = 0.0032, P = 0.0054, P = 0.0011.

D) Bar graph representing the rate of potassium efflux in activated Jurkat E6.1 T-cells under the indicated experimental conditions. From left to right P = 0.0547, P = 0.0052, P = 0.0017, P <0.0001.

E) Bar graph representing the mean intensity ratio of PBFI recorded in activated Jurkat E6.1 T-cells at 1200 seconds, as shown in Figures 1C and S1B. From left to right P = 0.0944, P = 0.1356, P = 0.0162, P = 0.0012)

F) Schematic representation summarizing the effect of elevated $[K^+]_e$ or clofazimine on K^+ efflux and Ca^{2+} influx in activated T cells. Black solid or dotted arrows indicate the pathways connecting TCR stimulation to K^+ efflux and Ca^{2+} influx, respectively. Red dotted or solid blunt arrows indicate the pathway inhibited in the presence of elevated $[K^+]_e$ or clofazimine treatment.

In each experimental series, Jurkat E6.1 T cells at the same passage were activated (on the same day) under the indicated experimental conditions. Statistical analysis of a two-tailed Student's t-test was performed. In the bar plots (panel C-E), the data represent mean $\pm$ SD (ns = not significant; *P<0.05; **P<0.01; ***P<0.001; ****P<0.0001). All data were plotted using GraphPad Prism version 9.5.1.

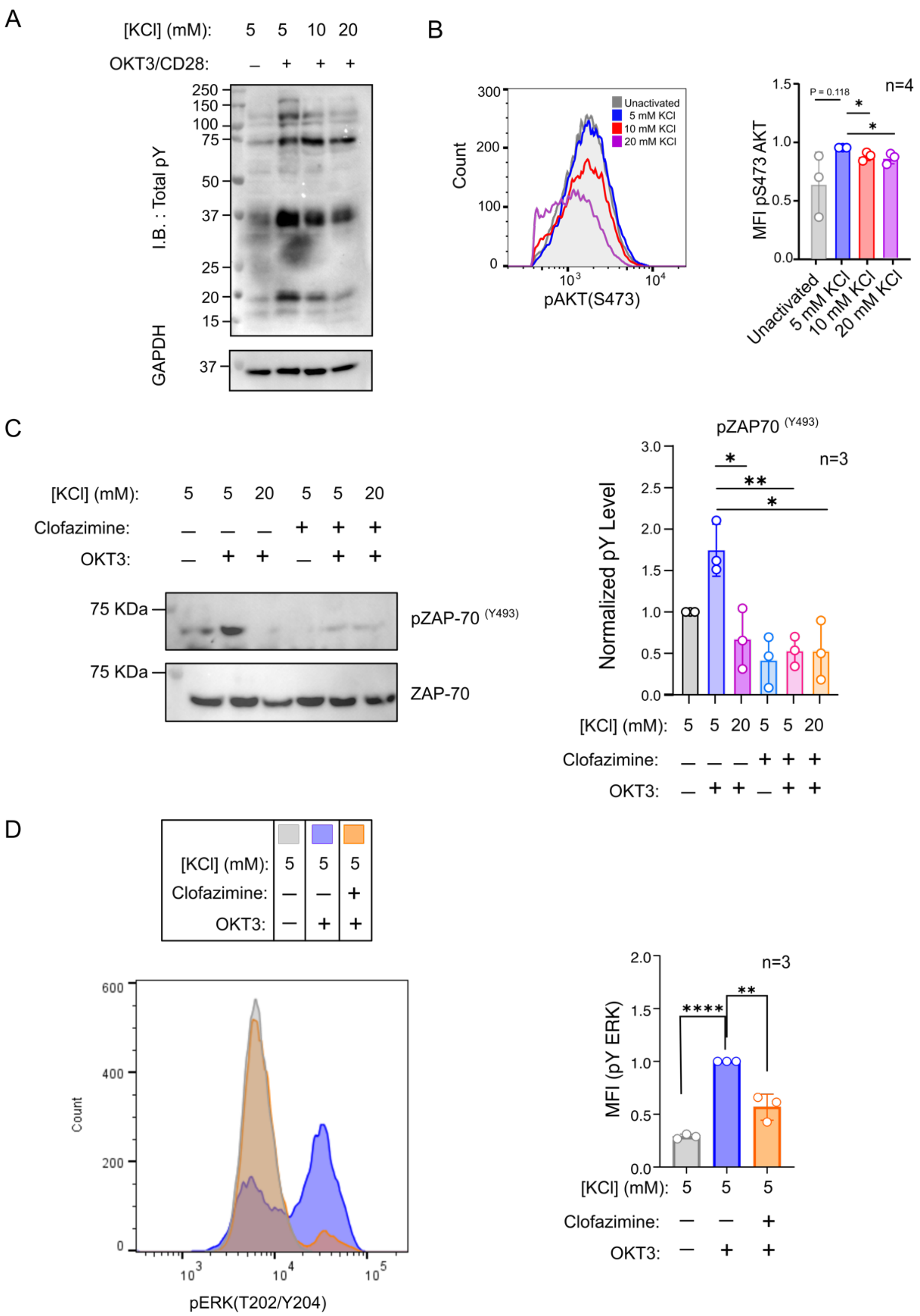
A
[KCl] (mM): 5 5 10 20
OKT3/CD28: – + + +
I.B. : Total pY
250 150 100 75 50 37 25 20 15
GAPDH
37
B
Count
pAKT(S473)
Unactivated
5 mM KCl
10 mM KCl
20 mM KCl
MFI pS473 AKT
P = 0.118
n=4
Unactivated
5 mM KCl
10 mM KCl
20 mM KCl
C
[KCl] (mM): 5 5 20 5 5 20
Clofazimine: – – – + + +
OKT3: – + + – + +
75 KDa
pZAP-70 (Y493)
ZAP-70
pZAP70 (Y493)
n=3
Normalized pY Level
D
[KCl] (mM): 5 5 5
Clofazimine: – – +
OKT3: – + +
Count
pERK(T202/Y204)
n=3
MFI (pY ERK)

Figure S2: Clofazimine treatment attenuates ZAP-70 activation following TCR stimulation.

A) Representative immunoblot analysis of total phosphotyrosine levels in Jurkat E6.1 T-cells in the indicated experimental conditions. Anti-GAPDH mAb staining is the loading control.

B) Representative flow cytometry histograms (on the left) of phospho-AKT (S473) in stimulated Jurkat E6.1 T-cells at the indicated extracellular potassium concentration. Adjacent bar graphs (on the right) show the fold change in AKT phosphorylation at the indicated extracellular potassium concentration (n=3). From left to right, P=0.118; P= 0.0268; P= 0.0286.

C) Representative immunoblot analysis of ZAP-70 activation in Jurkat E6.1 T-cells at the indicated experimental conditions. The cells were activated with OKT3/anti-CD28 for 5mins at 37^{0}C. The ZAP-70 activation was determined using ZAP-70-specific anti-pY493 mAb and anti-ZAP70 mAb used as a loading control. The bar graph (on the right) shows the relative phosphorylation level of ZAP-70 Y493 at the indicated experimental conditions (n=3). From left to right, P=0.0182; P=0.0043; P=0.0115.

D) Representative flow cytometry histogram of phosphor-Erk 1/2 (T202/Y204) in Jurkat E6.1 T cells under the indicated experimental conditions. The right panel shows the bar graph indicating the fold changes in Erk 1/2 (T202/Y204) phosphorylation measured by the flow cytometry experiment. From left to right, P<0.0001; P=0.0036;.

Statistical analysis of a two-tailed Student's t-test was performed. Central values and error bars in the bar plot represents mean $\pm$ SD (ns= not significant; *P<0.05; **P<0.01; ***P<0.001; ****P<0.0001). All data were plotted using GraphPad PrismVer 9.5.1. The flow cytometry data were analyzed using FlowJo Ver8. The schematics and icons were made using Inkscape Ver1.4.

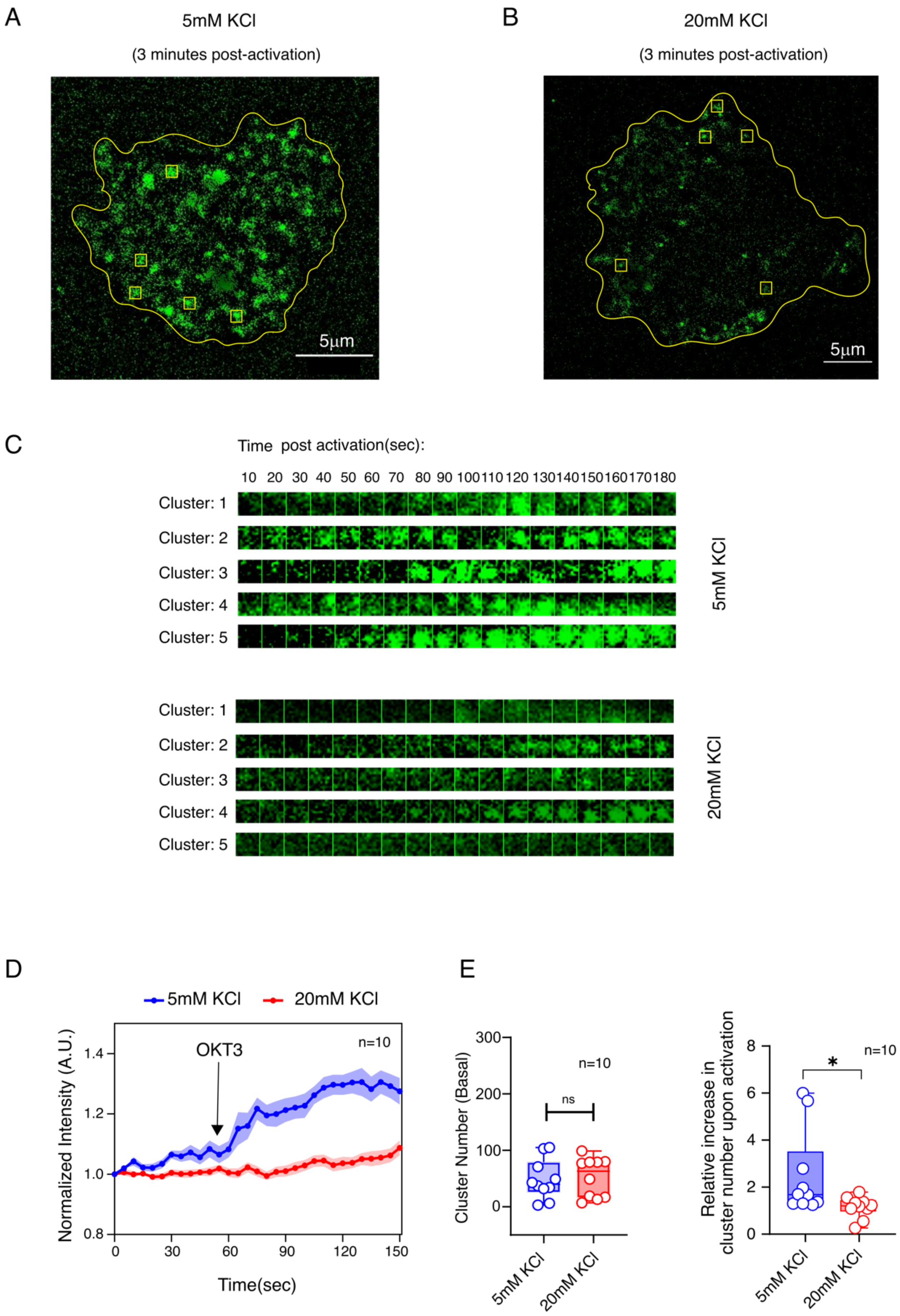
A
5mM KCl
(3 minutes post-activation)
5μm
B
20mM KCl
(3 minutes post-activation)
5μm
C
Time post activation(sec):
10 20 30 40 50 60 70 80 90 100 110 120 130 140 150 160 170 180
Cluster: 1
Cluster: 2
Cluster: 3
Cluster: 4
Cluster: 5
5mM KCl
Cluster: 1
Cluster: 2
Cluster: 3
Cluster: 4
Cluster: 5
20mM KCl
D
5mM KCl
20mM KCl
Normalized Intensity (A.U.)
OKT3
n=10
Time(sec)
E
Cluster Number (Basal)
n=10
ns
5mM KCl
20mM KCl
Relative increase in cluster number upon activation
*
n=10
5mM KCl
20mM KCl

Figure S3: Membrane recruitment of ZAP-70 by TIRF microscopy

A) and B) Representative live-cell images of Jurkat P116 T-cell showing the recruitment of the indicated ZAP-70 construct at three minutes post-activation with OKT3/anti-CD28 in the presence of indicated extracellular KCl. The cell boundary is marked with a yellow line, and the yellow box indicates five random clusters. The representative images are the same as in the right panel of Figure 3C.

C) Time-lapse montage of five ZAP-70 clusters formed after activating with OKT3/CD28 mAb in Jurkat P116 cells stably expressing ZAP-70-EGFP in the presence of 5mM $[K^+]_e$ (top panel), and 20mM (bottom panel) $[K^+]_e$.

D) The plot of mean EGFP intensity as a function of time measured from ten Jurkat P116 cells stably expressing the ZAP-70-EGFP construct. The arrow indicates the time point at which OKT3/anti-CD28 mAb is added. Each point represents the mean of the average intensity of five ZAP-70 clusters (size: 10 pixels, 1pixel= 0.65μm) from each cell (n=10 cells), and the error bar denotes the std error of the mean. The red and blue solid lines serve as guidance.

E) Box plot on the left shows the ZAP-70-EGFP cluster number per Jurkat P116 T cell measured at the basal state in the presence of 5 or 20 mM KCl. The box plot on the right shows the relative increase in the number of clusters in P116 cells stably expressing ZAP-70-EGFP at 3 minutes post-activation with OKT3/anti-CD28 (n=10 cells). P=0.034.

A statistical analysis of two-tailed Students' t-tests was performed. Data in panel E represents mean $\pm$ SD (ns= not significant; *P<0.05; **P<0.01; ***P<0.001; ****P<0.0001). The data in panel E is plotted using GraphPad PrismVer9.5.1. The image analysis was done using Fiji Ver 1.54m. The schematics and icons were made using Inkscape Ver1.4.

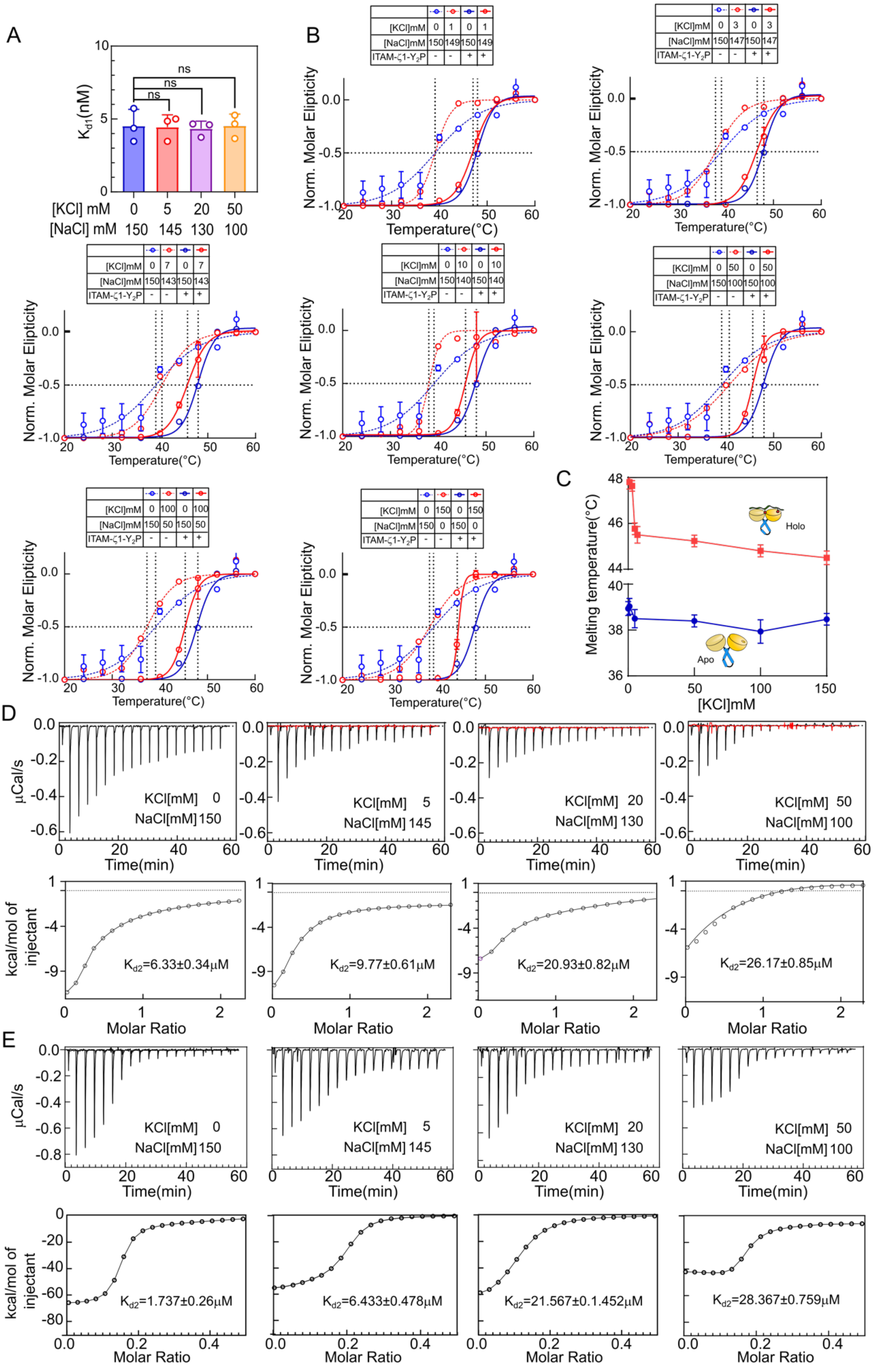

A
B
C
D
E
Kd1(nM)
[KCl] mM 0 5 20 50
[NaCl] mM 150 145 130 100
ns
Norm. Molar Elipticity
Temperature(°C)
ITAM-ζ1-Y2P
Melting temperature(°C)
[KCl]mM
Holo
Apo
μCal/s
Time(min)
kcal/mol of injectant
Molar Ratio
KCl[mM] 0 NaCl[mM] 150
KCl[mM] 5 NaCl[mM] 145
KCl[mM] 20 NaCl[mM] 130
KCl[mM] 50 NaCl[mM] 100
Kd2=6.33±0.34μM
Kd2=9.77±0.61μM
Kd2=20.93±0.82μM
Kd2=26.17±0.85μM
Kd2=1.737±0.26μM
Kd2=6.433±0.478μM
Kd2=21.567±0.1.452μM
Kd2=28.367±0.759μM

Figure S4: Potassium prevents ITAM-ζ1-Y_2P and ZAP-70 tSH2 interaction in a concentration dependent manner

A) The bar graph represents the dissociation constant (K_{d1}) for the ITAM-ζ1-Y_2P binding to the C-SH2 phosphate-binding pocket. The K_{d1} was obtained from the titration of ITAM-ζ1-Y_2P and ^{R39A}tSH2 domains by fluorescence polarization experiment. From left to right, P=0.5628, P=0.9365, P=0.1531.

B) Thermal denaturation profiles of *apo* (broken lines) and *holo* (solid line) ZAP-70 tSH2 domain were measured using CD spectroscopy at the indicated potassium concentration. The lines represent the fitting to the Boltzmann sigmoidal equation. The intersect between the black-dotted vertical and horizontal lines indicates the T_m.

C) Melting temperature (T_m) for the *apo* (blue circle) and *holo* (orange circle) tSH2 domain of ZAP-70 measured at increasing KCl concentration is plotted. The melting temperature is derived from the thermal denaturation profile measured from CD spectroscopy (n=3).

D) ITC titration of ITAM-ζ1-Y_2P and ZAP-70 tSH2 construct bearing R190A mutation. For each titration, 20 µM of tSH2 was titrated with 300 µM of ITAM-ζ1-Y_2P. *Top panel*: Black lines represent protein and ligand titration, and the *red line* represents buffer-to-buffer titration. *Bottom panel*: The *solid line* represents the fitting to the one-site binding model.

E) Representative ITC titration profile of the ITAM-ζ1-Y_2P and wild-type ZAP-70 tSH2 domain. For each titration, 20 µM of tSH2 was titrated with 66 µM of ITAM-ζ1-Y_2P. *Top panel*: Black lines represent protein and ligand titration, and the *red line* represents buffer-to-buffer titration. *Bottom panel*: The *solid line* represents the fitting to the sequential two-site binding model. The K^*_{d1} was fixed at 91 nM, 118 nM, 122 nM and 129 nM for the 0 mM, 5 mM, 20 mM, and 50 mM KCl concentration, respectively.

A statistical analysis of two-tailed Students' t-tests was performed. Each data point represent mean ± SD (ns= not significant; *P<0.05; **P<0.01; ***P<0.001; ****P<0.0001). All data were plotted using GraphPad PrismVer9.5.1. Fitting of the ITC data was done using Malvern PEAQ ITC Analysis Software (v 1.41). The schematics and icons were made using Inkscape Ver1.4.

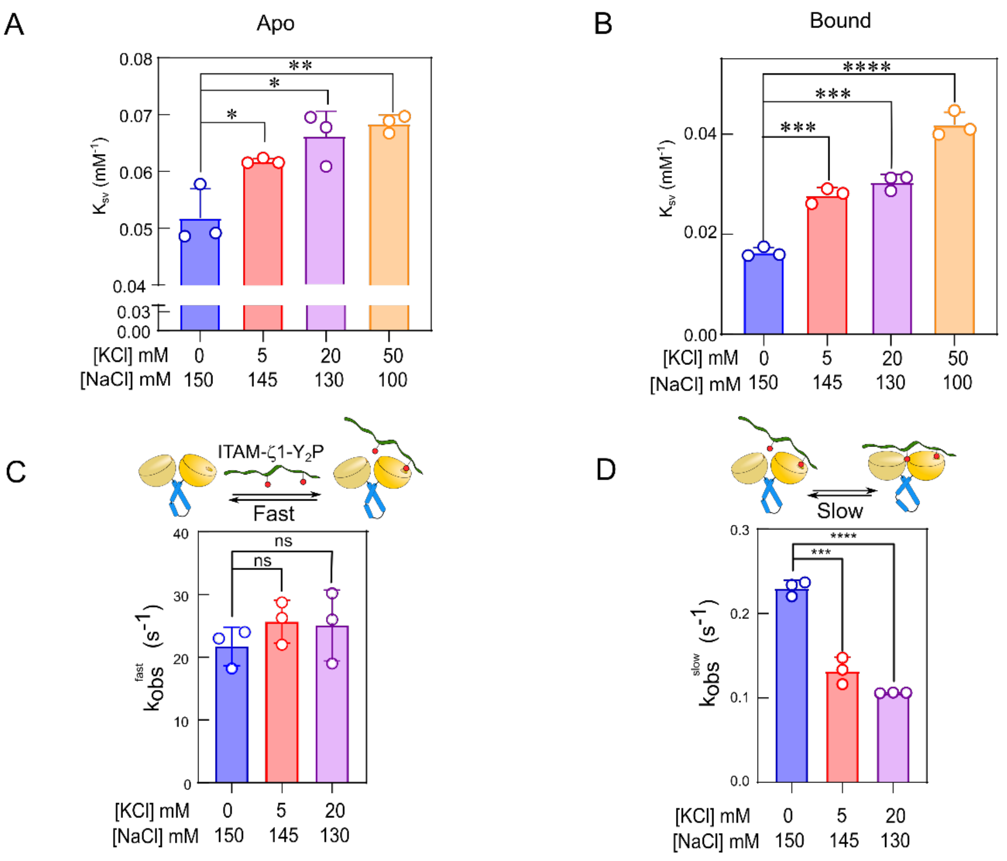

Figure S5: High potassium reduces the rate of structural transition of tSH2 domains from an open to a closed state

A) and B) The bar graph represents the Stern-Volmer quenching constant (K_{sv}) for the *apo* and *holo* tSH2 domain of ZAP-70 at the indicated salt concentration, respectively (n=3). From left to right *P*=0.0284, *P*=0.0230, *P*=0.0058, *P*=0.0004, *P*=0.0002, *P*=<0.0001.

C) and D) The bar graph represents the rate of change in intrinsic tryptophan fluorescence (K_{obs}^{fast} and K_{obs}^{slow}) of the tSH2 domain upon ITAM-ζ1-Y_2P binding at the indicated salt concentration. The K_{obs}^{fast} and K_{obs}^{slow} are obtained from the pre-steady state experiments described in Figure 6D and F. (n=3). From left to right *P*=0.2125, *P*=0.4207, *P*<0.0001, *P*=0.0007.

Panels A- D, a statistical analysis of two-tailed Students' t-tests was performed. Each data point represents mean ± SD (ns= not significant; **P*<0.05; ***P*<0.01; ****P*<0.001; *****P*<0.0001). All data were plotted using GraphPad PrismVer9.5.1. The schematics and icons were made using Inkscape Ver1.4.

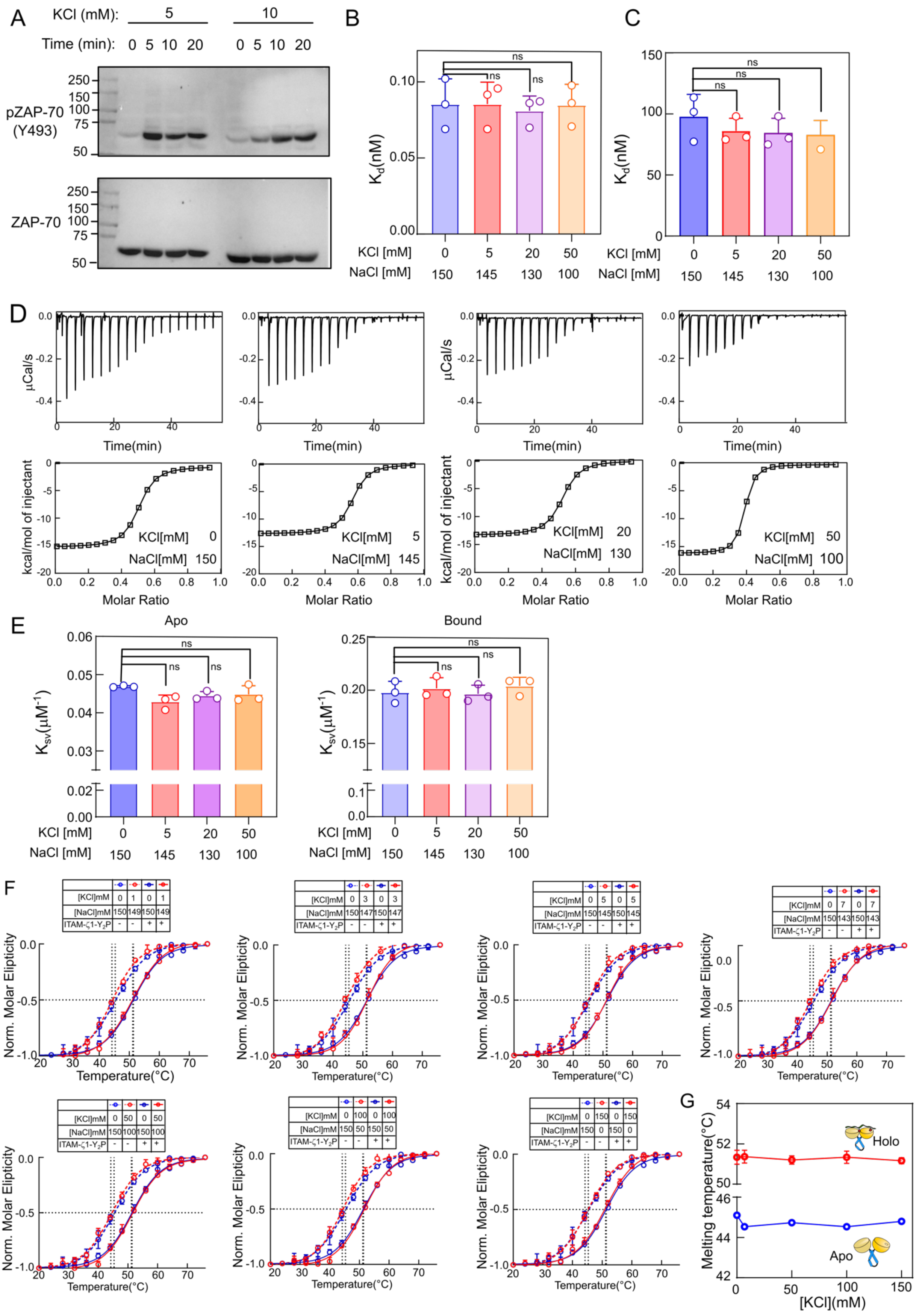

A
KCl (mM):
5
10
Time (min):
0 5 10 20
0 5 10 20
pZAP-70 (Y493)
ZAP-70
250
150
100
75
50
B
C
ns
K_d(nM)
KCl [mM]
NaCl [mM]
0 5 20 50
150 145 130 100
D
μCal/s
Time(min)
kcal/mol of injectant
Molar Ratio
KCl[mM] 0
NaCl[mM] 150
KCl[mM] 5
NaCl[mM] 145
KCl[mM] 20
NaCl[mM] 130
KCl[mM] 50
NaCl[mM] 100
E
Apo
Bound
K_{sv}(μM^{-1})
F
[KCl]mM
[NaCl]mM
ITAM-ζ1-Y_2P
Norm. Molar Elipticity
Temperature(°C)
G
Melting temperature(°C)
Holo
Apo
[KCl](mM)

Figure S6: Characterization of Syk tSH2 domain and ITAM-Y2P interaction at elevated potassium concentration

A) A representative immunoblot of ZAP-70 Y493 autophosphorylation upon stimulating Jurkat cells in the presence of the indicated extracellular KCl concentration.

B) The bar graph represents the dissociation constant (K_d) for the ITAM-ζ1-Y2P binding to the Syk tSH2 domain at the indicated salt composition, measured by fluorescence polarization.

C) The bar graph shows the dissociation constant (K_d) measured from the change in the intrinsic tryptophan fluorescence of the Syk tSH2 domain during titrations with ITAM-ζ1-Y2P at the indicated salt composition.

D) ITC titration of ITAM-ζ1-Y2P and Syk tSH2 domain. For each titration, 20 µM of tSH2 was titrated with 66 µM of ITAM-ζ1-Y2P. Top panel: Black lines represent protein and ligand titration, and the red line represents buffer-to-buffer titration. Bottom panel: The solid line represents the fitting to the one-site binding model.

E) The bar graph represents the Stern-Volmer quenching constant (K_{sv}) for the *apo* and *holo* tSH2 domain of Syk at the indicated salt concentration, respectively (n=3).

F) Thermal denaturation profiles of the *apo* (broken lines) and *holo* (solid line) Syk tSH2 domain were measured by CD spectroscopy at the indicated potassium concentrations. The lines represent the fitting to the Boltzmann sigmoidal equation. The intersection between the black-dotted vertical and horizontal lines indicates the T_m.

G) The melting temperatures (T_m) of the *apo* (blue circle) and *holo* (orange circle) Syk tSH2 domains, measured at increasing KCl concentrations, are plotted. The melting temperature is derived from the thermal denaturation profile measured from CD spectroscopy (n=3).

Panels B,C and E, a statistical analysis of two-tailed Students' t-tests was performed. Each data point represents mean ± SD (ns= not significant; *P<0.05; **P<0.01; ***P<0.001; ****P<0.0001). All data were plotted using GraphPad PrismVer9.5.1. The schematics and icons were made using Inkscape Ver1.4.
